# Supplementary material for: Photoactivatable metabolic warheads enable precise and safe ablation of target cells in vivo
Source: Nat Commun. 2021 Apr 22;12:2369. doi: 10.1038/s41467-021-22578-2 (PMC8062536; doi:10.1038/s41467-021-22578-2)
Supplement: Supplementary file 1 — Supplementary Information [file 41467_2021_22578_MOESM1_ESM.pdf]

# Supplementary Information

**Photoactivatable metabolic warheads enable precise and safe ablation of target cells in vivo**

## Table of Contents

Supplementary Figures

Supplementary Tables

Supplementary Methods

Supplementary References

## Supplementary Figures

a

| Compound | $S^1$ (eV) | $T^2$ (eV) | $S^1$-$T^2$ (eV) |
|---|---|---|---|
| **2a** | 2.55 | 1.98 | 0.57 |
| **3a** | 2.88 | 1.43 | 1.45 |
| **4a** | 2.79 | 2.70 | 0.09 |
| **5a** | 2.02 | 1.55 | 0.46 |

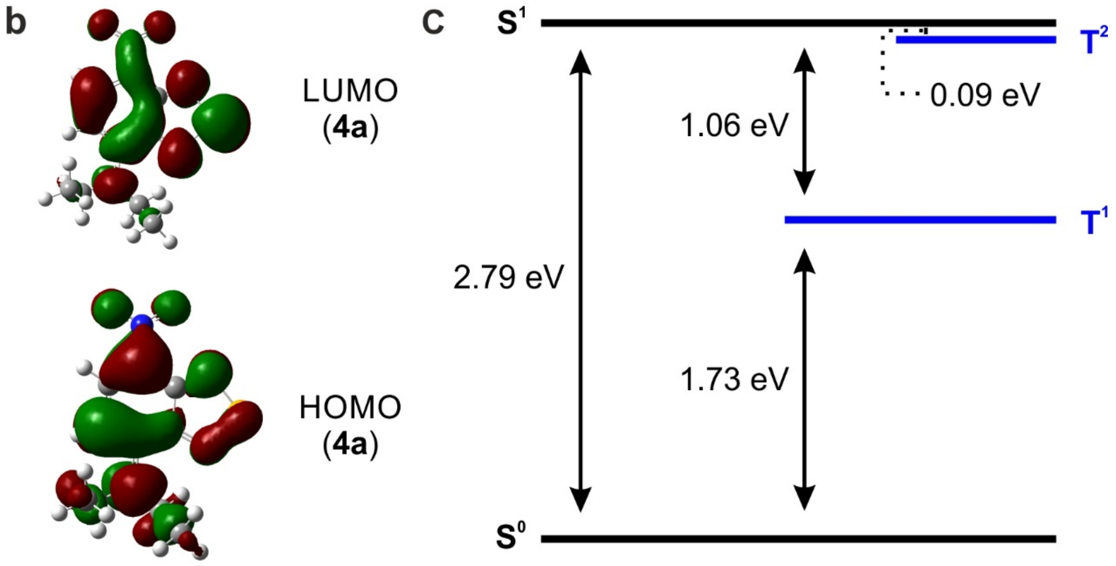


**Supplementary Figure 1**. **DFT calculations for the benzodiazole scaffolds 2a-5a**. (a) Energy values for both $S^1$ and $T^2$ levels and the $S^1$-$T^2$ intra-system crossing transition. Calculations performed using a B3YLP functional and def2tzvp basis set. (b) Molecular orbital diagrams of the HOMO and LUMO electron distributions of compound **4a**. (c) Jablonski diagram of the energy levels of compound **4a**.

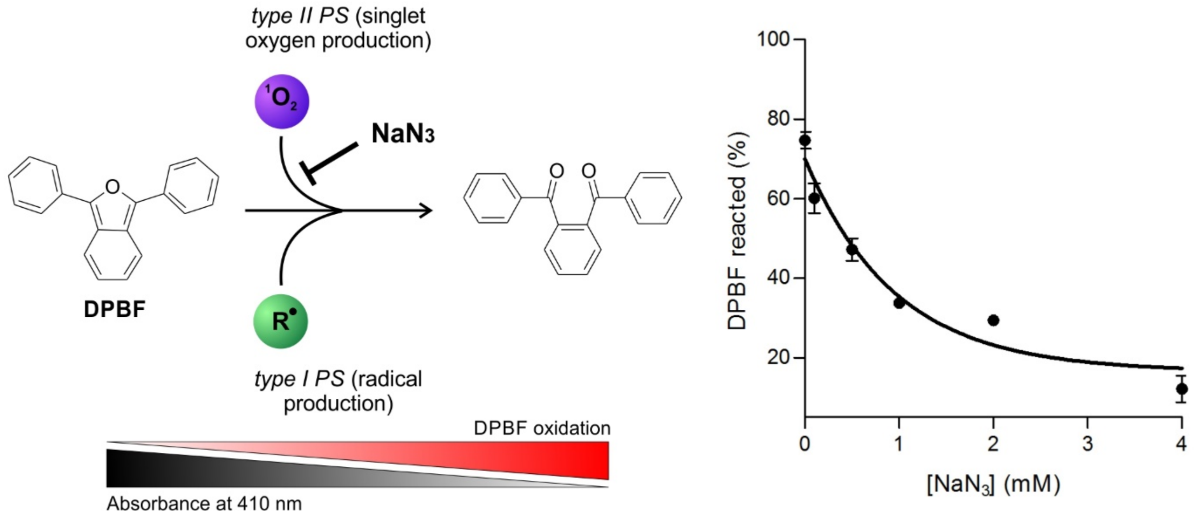


**Supplementary Figure 2. Evaluation of compound 4a as a type I/type II PS.** $NaN_3$ reacts selectively with $^1O_2$ produced by type II PS preventing the oxidation of 1,3-diphenylisobenzofuran (DPBF), whereas the oxidation of DPBF is not affected in type I PS. DPBF (400 μM) and **4a** (50 μM) were dissolved in EtOH (Note: DPBF was not soluble in water at these concentrations) and illuminated (0.35 mW, 520 nm, 1 h) in the absence and presence of sodium azide (0-4 mM). Ratios of DPBF oxidation were determined by normalization against absorbance at 410 nm in the absence of illumination. Data presented as mean values±SEM (n=4 independent experiments).

**a**

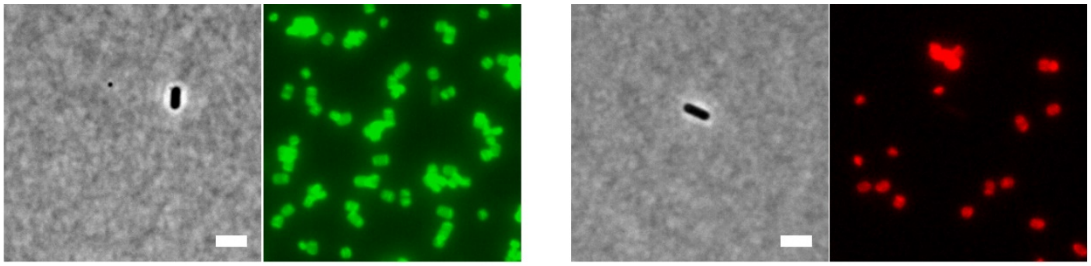

**NADA** **13**

**b**

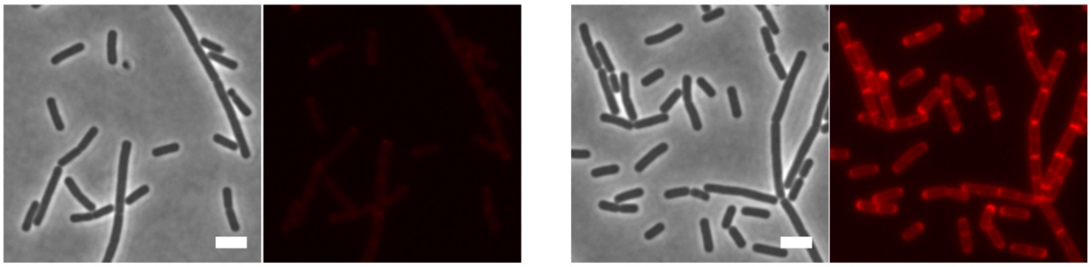

**12** **13**

**c**

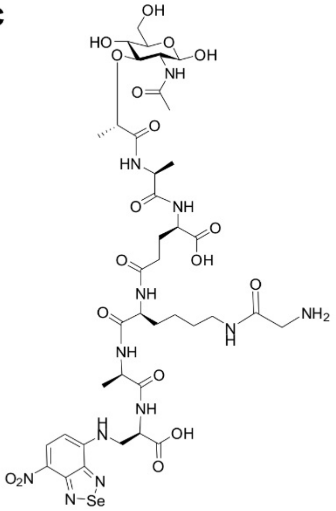


| PG-based structures including 13 | Calculated M+H$^+$ | Found M+H$^+$ |
|---|---|---|
| MurNAc-Ala-gln-(Lys-Gly)-ala-**13** | 1064.3235 | 1064.5208 |
| MurNAc-Ala-gln-Lys-ala-**13** | 1007.3021 | 1007.4995 |
| gln-Lys-ala-**13** | 660.1566 | 660.2976 |
| Lys-ala-**13** | 531.1141 | 531.7435 |
| **Other structures including 13** | **Calculated M+H$^+$** | **Found M+H$^+$** |
| Lys-[Gly$_2$]-ala-**13** | 645.1570 | 645.2945 |
| Lys-Gly-ala-**13** | 588.1355 | 588.2736 |
| Lys-[Gly$_7$]-ala-**13** | 859.2272 | 859.3998 |

**d**

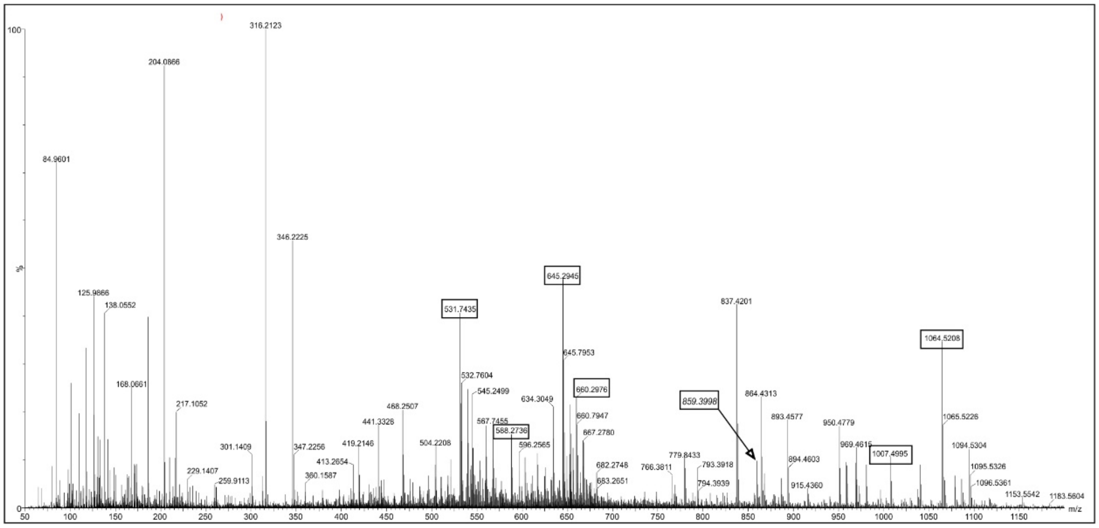

**Supplementary Figure 3. Selective incorporation of the amino acid 13 into bacterial cell walls.** (a) Representative phase and fluorescent microscopy images (from 2 independent experiments) of *E. coli* peptidoglycan sacculi isolated after the cells were grown in the presence of 100 µM compound **13** or **NADA**[1] for 2 h in LB medium at 37 °C. Similar to **NADA**, compound **13** covalently labels *E. coli* peptidoglycan cell walls ($\lambda_{exc}/_{em}$: 470/530 nm for **NADA** (green), $\lambda_{exc}/_{em}$: 508/590 nm for **13** (red)). Because isolated cell walls are typically not visible in the phase channel, the samples were spiked with unlabeled cells as fiducial markers. Scale bar: 1 µm. (b) Representative phase and fluorescent microscopy images (from 3 independent experiments) of *B. subtillis* incubated with compounds **12** or **13** (250 µM) and subsequently imaged under phase and fluorescence microscopy. Comparative incorporation of compounds **12** and **13** was imaged by fluorescence emission ($\lambda_{exc}/_{em}$: 508/590 nm) under the same image acquisition settings. Scale bar: 1 µm. (c) Chemical structure of a muropeptide with compound **13** incorporated at position 5. Table summarizing structures identified by MS/MS from extracts of *Staphylococcus aureus* cells upon growth in the presence of compound **13**. [$Gly_n$] indicate glycine bridges between the muropeptide stems. (d) MS spectrum for *S. aureus* muropeptides incorporating the amino acid **13**.

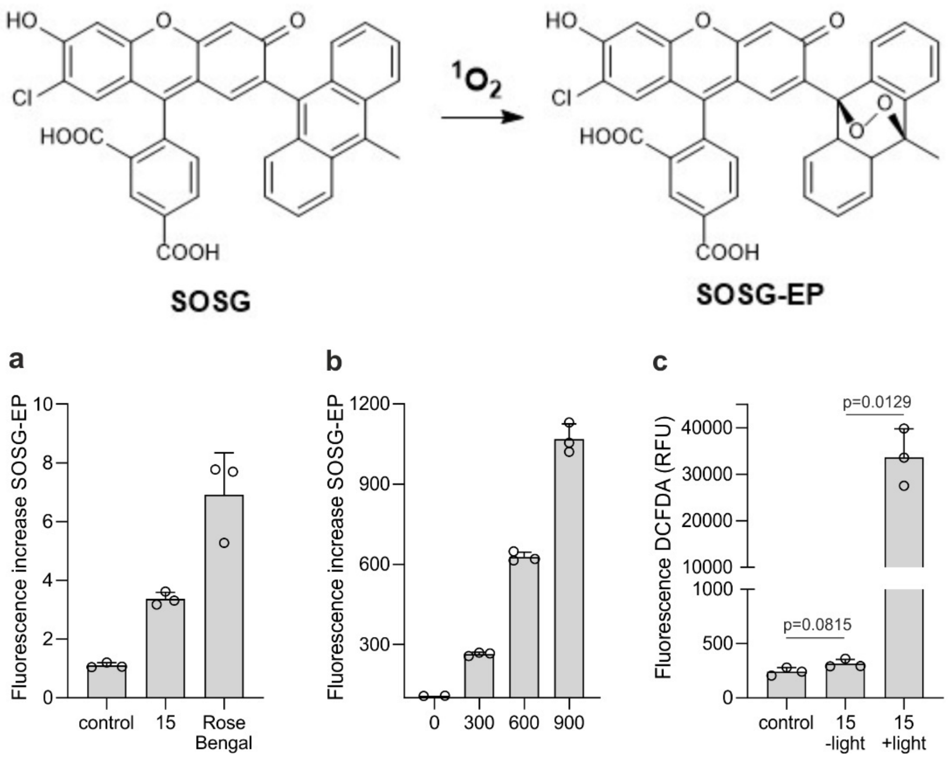


**Supplementary Figure 4. Singlet oxygen generation by compound 15 under single-photon and two-photon illumination.** Chemical structure of Singlet Oxygen Sensor Green (SOSG) and its fluorescent derivative (SOSG-EP) after reaction with singlet oxygen. (a) Singlet oxygen generation measured by fluorescence fold increase of SOSG (10 μM) in water after incubation with compound **15** (100 μM) and irradiation at 520 nm for 10 min; Rose Bengal (RB, 2.5 μM) was used as a positive control. Data presented as mean values±SD (n=3). (b) Fluorescence emission (520 nm) of aqueous solutions containing compound **15** (100 μM) and SOSG (10 μM) upon two-photon illumination at 970 nm with increasing power. Data presented as mean values±SD (n=3). (c) Flow cytometry of U87 cells after incubation with dichlorodihydrofluorescein diacetate (H2DCFDA) (20 μM) and compound **15** (100 μM) with or without illumination at 520 nm. Data presented as mean values±SD (n=3 independent experiments). P values from two-tailed unpaired t tests.

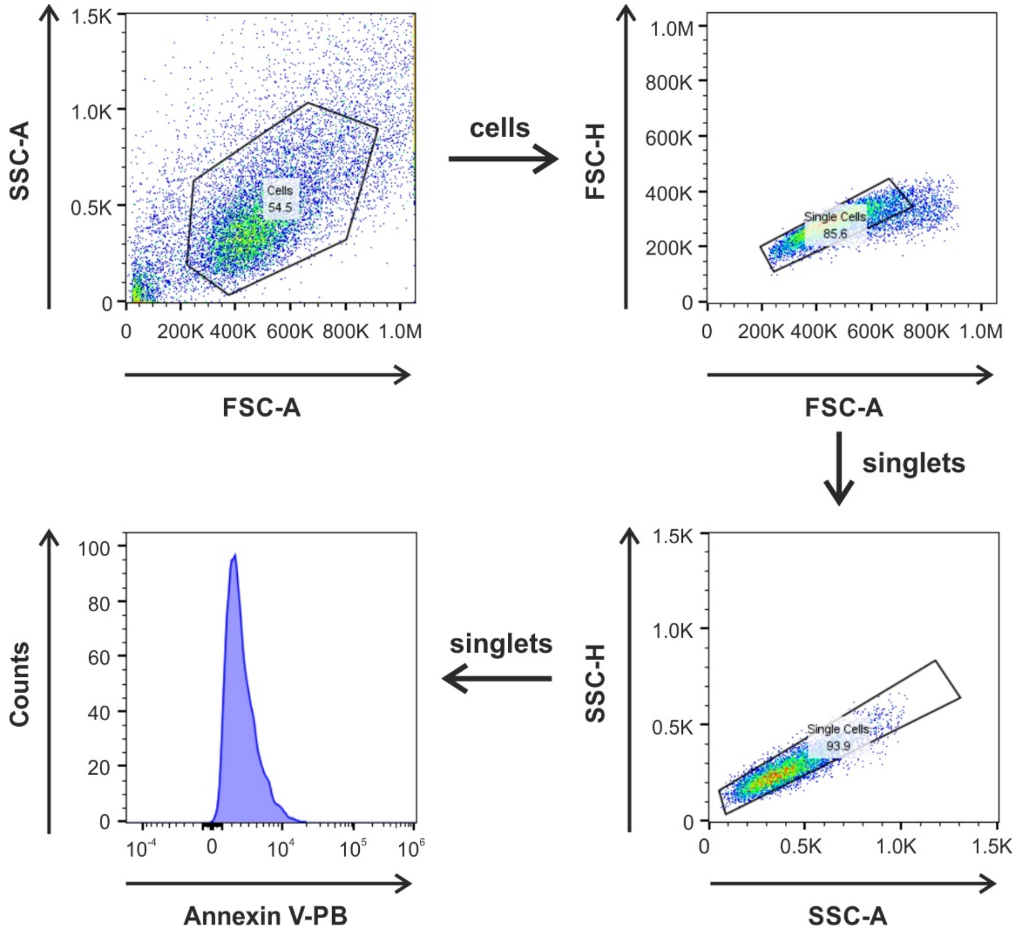


**Supplementary Figure 5. Gating strategy for U87 cells after different treatments with light and compound 15**. a) Gating strategy for the quantification of U87 cells after incubation with compounds **15-17** or AnnexinV-Pacific Blue and/or light irradiation. Representative plots from independent experiments performed in triplicate.

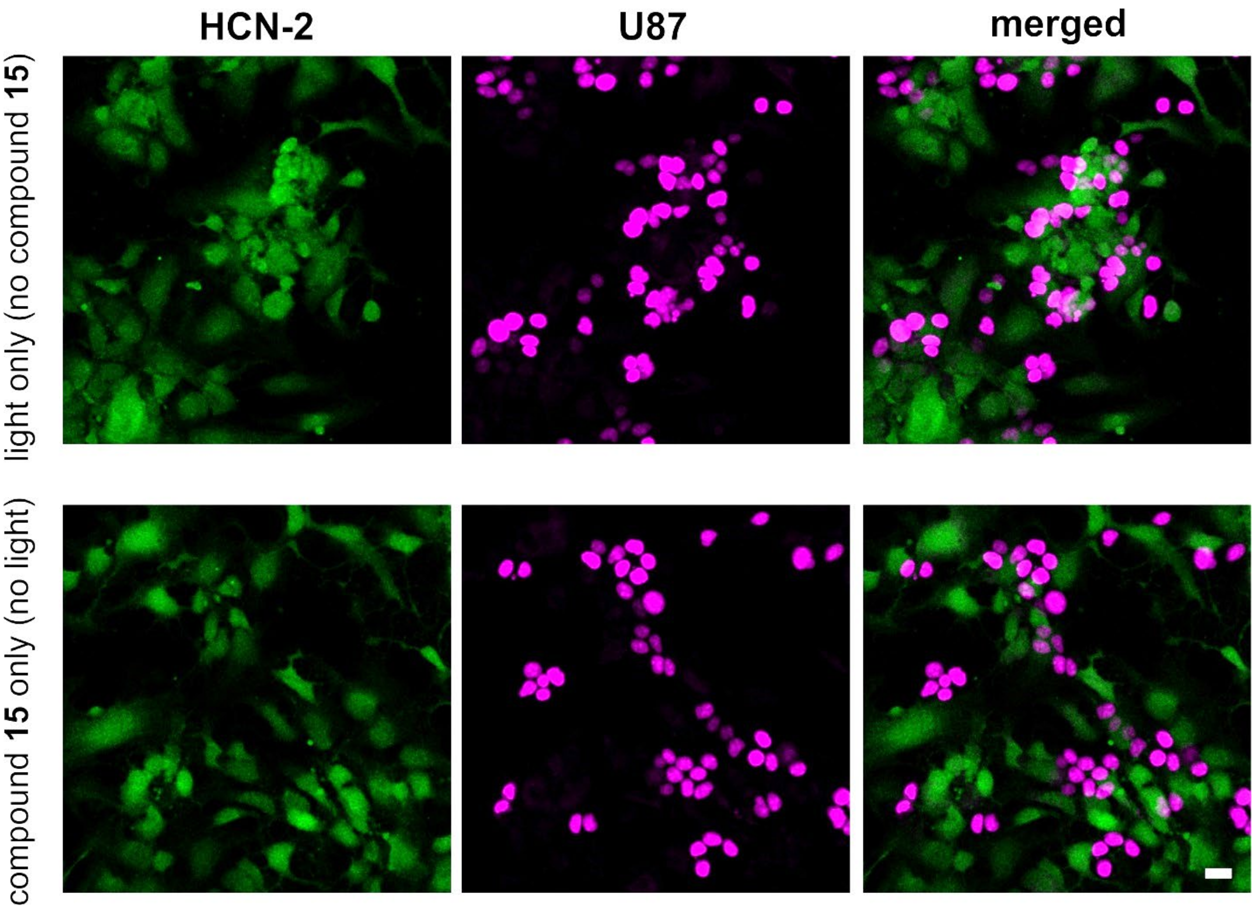


**Supplementary Figure 6. Compound 15 or light irradiation on their own do not kill U87 cells or HCN-2 cells in brain cell co-cultures.** Representative fluorescence confocal microscopy images (from 3 independent experiments) of human U87-nlsCrimson and HCN-2 co-cultures after light irradiation (37 J $cm^{-2}$) in the absence of compound **15** (top panels) or after incubation in the dark with compound **15** (100 μM) (bottom panels). Viable CellMask Green-labeled HCN-2 cells display green fluorescence emission ($\lambda_{exc}/_{em}$: 488/520 nm, green) and U87-nlsCrimson cells display far-red fluorescence emission ($\lambda_{exc}/_{em}$: 610/645 nm, magenta). Scale bar: 20 μm.

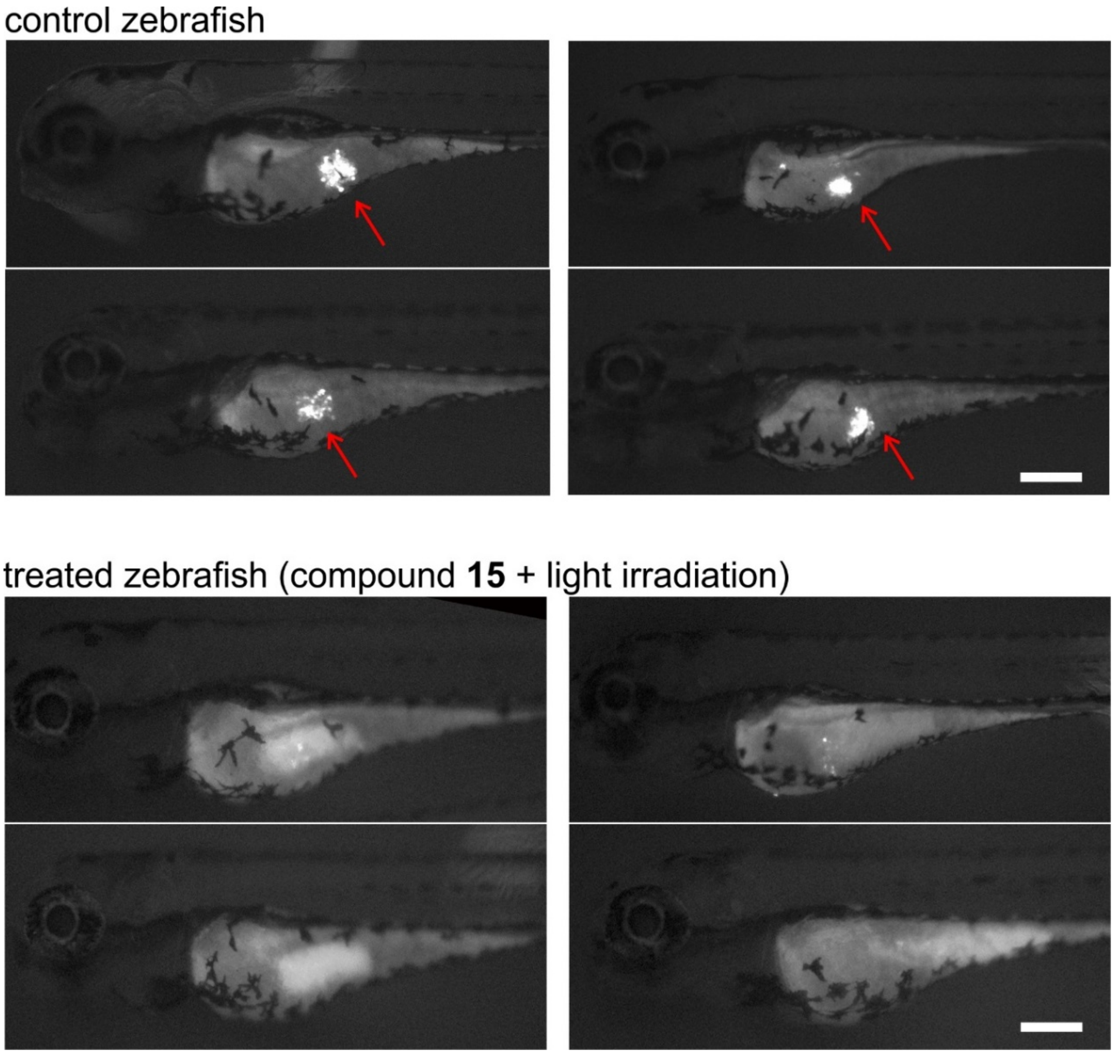


**Supplementary Figure 7. Combined treatment with compound 15 and light leads to removal of microtumors in vivo without changes in the morphology of zebrafish embryos.** Representative fluorescence confocal microscopy images of multiple zebrafish embryos (from 5 independent experiments) with human xenografts under physiological conditions and after injection of compound **15** (6 pg per zebrafish) followed by light illumination (10 mW, 37 J cm$^{-2}$). Red arrows point at tumor xenografts, where viable U87-nlsCrimson cells were imaged by far-red fluorescence emission ($\lambda_{exc}/_{em}$: 610/645 nm). Scale bar: 40 $\mu$m.

## Supplementary Tables

**Supplementary Table 1.** Chemical and spectral characterization for compounds **2a**-**17**.

| Code | HPLC purity | $M_{calc.}$ | $M_{exp.}$ | $\lambda_{abs.}$ (nm)[¥] | $\lambda_{em.}$ (nm)[¥] | $\Phi_{\Delta}$ (%)[‡] |
|---|---|---|---|---|---|---|
| **2a** | 99% | 236.1 | 236.2 | 490 | 540 | n.d. |
| **3a** | 99% | 253.1 | 253.2 | 480 | 545 | n.d. |
| **4a** | 99% | 323.0 | 323.1 | 510 | 610 | n.d. |
| **5a** | 93% | 263.1 | 263.1 | 565 | 650 | n.d. |
| **6** | 97% | 312.2 | 312.4 | 510 | 620 | n.d. |
| **7** | 99% | 352.0 | 352.0 | 400 | 450 | n.d. |
| **8** | 99% | 368.0 | 367.9 | 440 | 630 | n.d. |
| **9** | 96% | 355.0 | 355.0 | 425 | 610 | n.d. |
| **11a** | 93% | 426.9 | 426.8 | 500 | 650 | n.d. |
| **12** | 99% | 332.0 | 332.0 | 470 | 590 | 13±1 |
| **13** | 99% | 332.0 | 332.0 | 470 | 590 | 8±1 |
| **14** | 96% | 435.0 | 435.1 | 510 | 605 | 4.0±0.2 |
| **15** | 99% | 407.0 | 407.0 | 495 | 610 | 24±1 |
| **16** | 99% | 607.7 | 607.5 | 644 | 688 | <1 |
| **17** | 99% | 697.8 | 697.3 | 320 | 496 | 21[†] |

[¥] Absorbance and emission wavelengths in EtOH (concentration of compounds: 100 μM).

[‡] Singlet oxygen generation quantum yields were determined using DPBF in EtOH (Note: DPBF was found insoluble in water) using Rose Bengal as a reference.[2] Data presented as means±SEM (n = 3).

[†] Reported value for TPE using 9,10-anthrancenediylbis(mehtylen)diamalonic acid (ABDA) in water as a reference.[3]

**Supplementary Methods**

**Chemical synthesis**

**General materials**. Commercially available reagents were used without further purification. Thin-layer chromatography was conducted on Merck silica gel 60 F254 sheets and visualized by UV (254 and 365 nm). Silica gel (particle size 35–70 μm) was used for column chromatography. $^{1}H$ and $^{13}C$ spectra were recorded in a Bruker Avance 500 spectrometer (at 500 and 125 MHz, respectively). Data for $^{1}H$ NMR spectra are reported as chemical shift δ (ppm), multiplicity, coupling constant (Hz) and integration. Data for $^{13}C$ NMR spectra reported as chemical shifts relative to the solvent peak. HPLC-MS analysis was performed on a Waters Alliance 2695 separation module connected to a Waters PDA2996 photodiode array detector and a ZQ Micromass mass spectrometer (ESI-MS) with a Phenomenex® column ($C_{18}$, 5 μm, 4.6×150 mm). HPLC purifications were conducted using a Waters semipreparative HPLC system fitted with a Phenomenex column (C18 Axial, 10 μm, 21.2×150 mm) and UV detection.

***N*,*N*-diethyl-7-nitrobenzo[c][1,2,5] oxadiazol-4-amine (2a)**

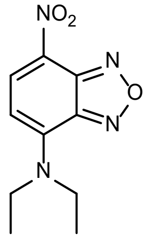


4-Chloro-7-nitrobenzo[c][1,2,5]oxadiazole (**NBD-Cl**, 20 mg, 0.08 mmol, 1 eq) was dissolved in EtOAc (1 mL). *N*,*N*-diethylamine (43 μL, 0.24 mmol, 3 eq) was then added and the reaction was stirred at r.t. for 5 min. Upon completion, volatiles were removed under reduced pressure and the crude product was purified by column chromatography (DCM) to give compound **2a** (24 mg, orange solid, quantitative yield).

$^1H$ NMR (500 MHz, MeOD) δ 8.50 (d, *J* = 9.2 Hz, 1H), 6.41 (d, *J* = 9.2 Hz, 1H), 4.05 (br, s, 4H), 1.41 (t, *J* = 7.1 Hz, 6H). $^{13}C$ NMR (125 MHz, MeOD) δ 145.1, 145.0, 144.5, 135.9, 101.3, 42.1, 10.1.

HRMS (m/z, ESI): calcd for $C_{10}H_{13}N_4O_3^+$ $[M+H]^+$: 237.0982, found: 236.0971.

**N,N-diethyl-7-nitrobenzo[c][1,2,5]thiadiazol-4-amine (3a)**

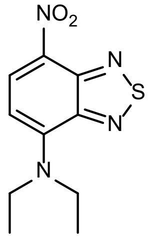

4-Fluoro-7-nitrobenzo[c][1,2,5]thiadiazole **3**[4] (20 mg, 0.08 mmol, 1 eq) was dissolved in MeCN (1 mL). Triethylamine (21 μL, 0.12 mmol, 1.5 eq) was then added as well as *N*,*N*-diethylamine (22 μL, 0.12 mmol, 1.5 eq) and reaction was stirred at r.t. for 5 min. Upon completion, volatiles were removed under reduced pressure and the crude product was purified by column chromatography (DCM:MeOH, 98:2) to give compound **3a** (24 mg, orange solid, 93%).

$^1H$ NMR (500 MHz, DMSO) δ 8.53 (d, *J* = 9.4 Hz, 1H), 6.65 (d, *J* = 9.4 Hz, 1H), 4.01 (q, *J* = 7.1 Hz, 4H), 1.32 (t, *J* = 7.1 Hz, 6H). $^{13}C$ NMR (125 MHz, DMSO) δ 150.2, 147.2, 145.2, 133.2, 126.3, 103.6, 48.0, 13.1.

HRMS (m/z, ESI): calcd for $C_{10}H_{13}N_4O_2S^+$ $[M+H]^+$: 253.0754, found: 253.0741.

***N,N*-diethyl-7-nitrobenzo[c][1,2,5]selenadiazol-4-amine (4a)**

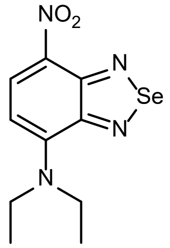


4-Fluoro-7-nitrobenzo[c][1,2,5]selenadiazole **4F**[4] (25 mg, 0.08 mmol, 1 eq) was dissolved in MeCN (1 mL). Triethylamine (21 μL, 0.12 mmol, 1.5 eq) was then added as well as *N*,*N*-diethylamine (22 μL, 0.12 mmol, 1.5 eq) and reaction was stirred at r.t. for 5 min. Upon completion, volatiles were removed under reduced pressure and the crude product was purified by column chromatography (DCM:MeOH 98:2) to give compound **4a** (21 mg, red solid, 70%).

$^1$H NMR (500 MHz, DMSO-$d_6$) δ 8.50 (d, $J$ = 9.4 Hz, 1H), 6.48 (d, $J$ = 9.4 Hz, 1H), 4.01 (q, $J$ = 7.0 Hz, 4H), 1.32 (t, $J$ = 7.0 Hz, 6H). $^{13}$C NMR (125 MHz, DMSO-$d_6$) δ 153.9, 152.4, 148.7, 134.1, 128.2, 101.9, 47.8, 13.2.

HRMS (m/z, ESI): calcd for $C_{10}H_{13}N_4O_2SeNa^+$ [M+H]$^+$: 301.0199, found: 301.0188.

***N,N*-diethyl-2,2-dimethyl-7-nitro-2*H*-benzo[d]imidazol-4-amine (5a)**

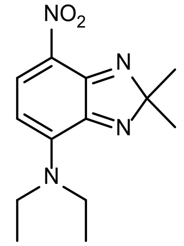


To a solution of 4-fluoro-2,2-dimethyl-7-nitro-2*H*-benzo[d]imidazole **5**[4] (10 mg, 0.05 mmol, 1 eq) in MeCN (1 mL), $NaHCO_3$ (11 mg, 0.13 mmol, 2.6 eq) in 0.5 mL $H_2O$ was added, followed by *N,N*-diethylamine (9 mL, 0.05 mmol, 1 eq) and the reaction was heated at 65 $^oC$ for 3h. The reaction mixture was then cooled down to r.t., acidified with 0.2 N HCl and extracted with EtOAc (2 × 20 mL). The organic layer was dried over anhydrous $MgSO_4$, the solvent was removed under reduced pressure and the crude product was purified by column chromatography (DCM:MeOH 95:5) to give compound **5a** (10 mg, purple solid, 80%).

$^1H$ NMR (500 MHz, MeOD) δ 8.46 (d, *J* = 9.4 Hz, 1H), 6.00 (d, *J* = 9.4 Hz, 1H), 4.06 (br, s, 4H), 1.61 (s, 6H), 1.35 (t, *J* = 7.1 Hz, 6H). $^{13}C$ NMR (125 MHz, MeOD) δ 155.3, 152.2, 150.6, 143.2, 124.4, 104.9, 99.4, 21.2, 11.3.

HRMS (m/z, ESI): calcd for $C_{13}H_{19}N_4O_2^+$ $[M+H]^+$: 263.1503, found: 263.1511.

**N-cyclohexyl-7-nitrobenzo[c][1,2,5]selenadiazol-4-amine (6)**

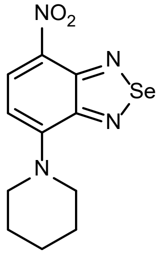


4-Fluoro-7-nitrobenzo[c][1,2,5]selenadiazole **4F** (20 mg, 0.08 mmol, 1 eq) was dissolved in MeCN (1 mL). Piperidine (24 μL, 0.24 mmol, 3 eq) was then added and reaction was stirred at r.t. for 5 min. Upon completion, volatiles were removed under reduced pressure and the crude product was purified by column chromatography (DCM:MeOH 99:1) to give compound **6** (15 mg, red solid, 59%).

$^{1}$H NMR (500 MHz, DMSO-$d_6$) δ 8.50 (d, $J$ = 9.1 Hz, 1H), 6.67 (d, $J$ = 9.2 Hz, 1H), 4.06 – 4.01 (m, 4H), 1.73 (s, 6H). $^{13}$C NMR (125 MHz, DMSO-$d_6$) δ 153.6, 153.3, 150.4, 133.4, 130.1, 104.8, 51.3, 26.3, 24.4.

HRMS (m/z, ESI): calcd for $C_{11}H_{13}N_4O_2Se^+$ $[M+H]^+$: 313.0199, found: 313.0186.

**4-(4-methoxyphenoxy)-7-nitrobenzo[c][1,2,5]selenadiazole (7)**

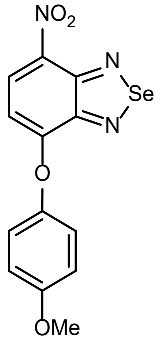


4-Fluoro-7-nitrobenzo[c][1,2,5]selenadiazole **4F** (20 mg, 0.08 mmol, 1 eq) was dissolved in MeCN (2 mL). 4-Methoxyphenol (12 mg, 0.08 mmol, 1 eq) was then added, followed by triethylamine (14 μL, 0.08 mmol, 1 eq) and reaction was stirred at r.t. for 1.5 h. Volatiles were removed under reduced pressure and the crude product was purified by column chromatography (Hexane:EtOAc 8:2 → 7:3) to give **7** (18 mg, orange solid, 63%).

$^{1}$H NMR (500 MHz, DMSO-$d_6$) δ 8.56 (d, $J$ = 8.5 Hz, 1H), 7.29 (d, $J$ = 9.1 Hz, 2H), 7.10 (d, $J$ = 9.1 Hz, 2H), 6.59 (d, $J$ = 8.5 Hz, 1H), 3.82 (s, 3H). $^{13}$C NMR (125 MHz, DMSO-$d_6$) δ 157.6, 156.8, 153.4, 152.3, 147.3, 135.5, 131.4, 122.5, 116.0, 106.8, 56.0.

HRMS (m/z, ESI): calcd for $C_{13}H_{10}N_3O_4Se^+$ [M+H]$^+$: 351.9832, found: 351.9827.

**4-((4-methoxyphenyl)thio)-7-nitrobenzo[c][1,2,5]selenadiazole (8)**

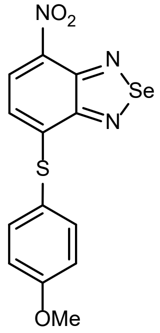


4-Fluoro-7-nitrobenzo[c][1,2,5]selenadiazole **4F** (20 mg, 0.08 mmol, 1 eq) was dissolved in MeCN (2 mL). 4-Methoxythiophenol (10 μL, 0.08 mmol, 1 eq) was then added, followed by triethylamine (14 μL, 0.08 mmol, 1 eq). A red precipitate was immediately formed, and after 5 min stirring at r.t. it was collected by filtration and washed with MeCN to give the expected product **8** (25 mg, red solid, 83%).

$^1$H NMR (500 MHz, DMSO-$d_6$) δ 8.39 (d, $J$ = 8.1 Hz, 1H), 7.65 (d, $J$ = 8.1 Hz, 2H), 7.20 (d, $J$ = 8.1 Hz, 2H), 6.64 (d, $J$ = 8.1 Hz, 1H), 3.87 (s, 3H). $^{13}$C NMR (125 MHz, DMSO-$d_6$) δ 161.6, 156.8, 150.4, 146.7, 137.9, 137.6, 128.8, 119.5, 118.1, 116.7, 56.0.

HRMS (m/z, ESI): calcd for $C_{13}H_{10}N_3O_3SSe^+$ $[M+H]^+$: 367.9602, found: 367.9613.

**4-(4-methoxyphenyl)-7-nitrobenzo[c][1,2,5]selenadiazole (9)**

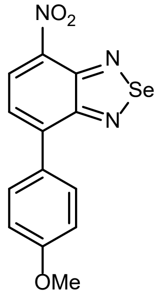


4-Chloro-7-nitrobenzo[c][1,2,5]selenadiazole **4Cl**[4] (20 mg, 0.08 mmol, 1 eq) and 4-methoxyphenylboronic acid (12 mg, 0.08 mmol, 1 eq) were placed in a round bottom flask fitted with a reflux condenser under a $N_2$ atmosphere. Dioxane (2 mL) was then added via a syringe and the mixture was stirred, followed by addition of $CsCO_3$ (74 mg, 0.24 mmol, 3 eq) and $Pd(PPh_3)_4$ (4 mg, 20% wt.). Reaction was then heated at 100 °C for 2 h. It was then cooled down to r.t., diluted with brine (25 mL) and extracted with EtOAc (3 × 25 mL). The organic layer was dried over anhydrous $MgSO_4$, the solvent was removed under reduced pressure and the crude product was purified by column chromatography (DCM:Hexane 1:1 → DCM) to give **9** (3 mg, orange solid, 10%).

$^1$H NMR (500 MHz, DMSO-$d_6$) δ 8.57 (d, $J$ = 7.8 Hz, 1H), 7.97 (d, $J$ = 8.9 Hz, 2H), 7.80 (d, $J$ = 7.8 Hz, 1H), 7.14 (d, $J$ = 8.9 Hz, 2H), 3.87 (s, 3H). $^{13}$C NMR (125 MHz, DMSO-$d_6$) δ 160.8, 159.2, 151.6, 141.5, 139.6, 135.0, 131.9, 129.4, 128.8, 128.3, 124.9, 114.5, 55.8. HRMS (m/z, ESI): calcd for $C_{13}H_{10}N_3O_3Se^+$ $[M+H]^+$: 334.9828, found: 334.9838.

**4-fluoro-6-iodo-7-nitrobenzo[c][1,2,5]selenadiazole (11)**

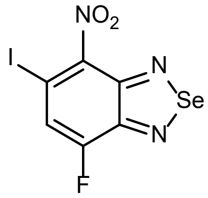


$SeO_2$ (44 mg, 0.6 mmol, 1.5 eq) was added to a solution of 3-fluoro-5-iodobenzene-1,2-diamine **10** (100 mg, 0.4 mmol, 1 eq) in EtOH (2 mL) and the reaction was refluxed for 3 h. Upon completion, the solvent was removed under reduced pressure and the crude product was quickly purified by column chromatography (DCM) to give 4-fluoro-6-iodobenzo[c][1,2,5]selenadiazole as a light yellow solid that was redissolved in concentrated $H_2SO_4$ (2.5 mL) and concentrated $HNO_3$ (1 mL) and stirred for 2 h at r.t. Product was then precipitated upon addition of the reaction mixture into cold $H_2O$ (20 mL) and purified by column chromatography (DCM) to give **11** in ~65% purity (~35% of non-nitrated intermediate) and was used in the next step without further purification.

$^1$H NMR (500 MHz, DMSO-$d_6$) δ 7.94 (d, $J_{H\text{-}F}$ = 9.7 Hz, 1H).

HRMS (m/z, ESI): calcd for $C_6H_2FIN_3O_2Se^+$ $[M+H]^+$: 371.8190, found: 371.8212.

**_N_,_N_-diethyl-6-iodo-7-nitrobenzo[c][1,2,5]selenadiazol-4-amine (11a)**

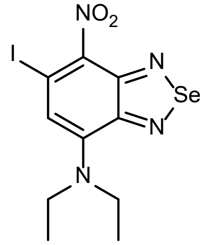

4-Fluoro-6-iodo-7-nitrobenzo[c][1,2,5]selenadiazole **11** (30 mg, 0.08 mmol, 1 eq) was dissolved in MeCN (2 mL). *N,N*-diethylamine (28 μL, 0.16 mmol, 2 eq) was then added and reaction was stirred at r.t. for 3 h. Upon completion, volatiles were removed under reduced pressure and the crude product was purified by column chromatography (DCM:Hexane 1:1) to give **11a** (11 mg, red solid, 55%).

$^{1}$H NMR (500 MHz, DMSO-$d_6$) δ 6.65 (s, 1H), 3.89 (q, $J$ = 7.0 Hz, 4H), 1.26 (t, $J$ = 7.0 Hz, 6H). $^{13}$C NMR (125 MHz, DMSO-$d_6$) δ 153.2, 151.8, 144.0, 137.1, 112.1, 98.9, 47.0, 13.1. HRMS (m/z, ESI): calcd for $C_{10}H_{11}IN_4O_2Se^-$ [M]$^-$: 425.9228, found: 425.9055.

**(*S*)-2-amino-3-((7-nitrobenzo[c][1,2,5]selenadiazol-4-yl)amino) propanoic acid hydrochloride (12)**

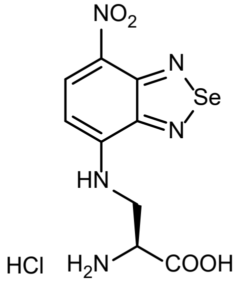


A solution of Boc-L-2,3-diaminopropionic acid (9 mg, 0.044 mmol, 1.1 eq) and $NaHCO_3$ (10 mg, 0.12 mmol, 3 eq) in water (0.4 mL) was heated at 55 ºC. A solution of 4-fluoro-7-nitrobenzo[c][1,2,5]selenadiazole **4F** (10 mg, 0.04 mmol, 1 eq) in MeOH (1 mL) was then added dropwise and the reaction was kept stirring at 55 ºC for 1 h. The solvent was removed under reduced pressure and the crude product was quickly purified by column chromatography (DCM:MeOH 9:1) to give *N*-Boc-(*S*)-2-amino-3-((7-nitrobenzo[c][1,2,5] selenadiazol-4-yl)amino)propanoic acid hydrochloride as a red solid, that was redissolved in 4 N HCl in dioxane (6 mL) and stirred for 30 min at r.t. to give **12** (15 mg, red solid, quantitative yield over two steps) upon removal of the solvent.

$^{1}H$ NMR (500 MHz, DMSO-$d_6$) δ 8.62 (d, $J$ = 8.8 Hz, 1H), 8.54 (br, s, 2H), 8.27 (br, t, $J$ = 5.5 Hz, 1H), 6.56 (d, $J$ = 8.9 Hz, 1H), 4.22 (t, $J$ = 5.8 Hz, 1H), 4.13 – 3.87 (m, 2H). $^{13}C$ NMR (125 MHz, DMSO-$d_6$) δ 169.5, 152.5, 152.1, 148.2, 135.2, 129.7, 98.4, 51.7, 43.2. HRMS (m/z, ESI): calcd for $C_9H_{10}N_5O_4Se^+$ $[M+H]^+$: 331.9892, found 331.9895.

**(*R*)-2-amino-3-((7-nitrobenzo[c][1,2,5]selenadiazol-4-yl)amino) propanoic acid hydrochloride (13)**

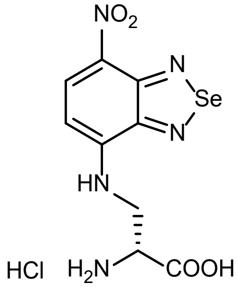


A solution of Boc-D-2,3-diaminopropionic acid (9 mg, 0.044 mmol, 1.1 eq) and $NaHCO_3$ (10 mg, 0.12 mmol, 3 eq) in water (0.4 mL) was heated at 55 ºC. A solution of 4-fluoro-7-nitrobenzo[c][1,2,5]selenadiazole **4F** (10 mg, 0.04 mmol, 1 eq) in MeOH (1 mL) was then added dropwise and the reaction was kept stirring at 55 °C for 1 h. The solvent was removed under reduced pressure and the crude product was quickly purified by column chromatography (DCM:MeOH 9:1) to give *N*-Boc-(*R*)-2-amino-3-((7-nitrobenzo[c][1,2,5] selenadiazol-4-yl)amino)propanoic acid hydrochloride as a red solid, that was redissolved in 4 N HCl in dioxane (6 mL) and stirred for 30 min at r.t. to give **13** (15 mg, red solid, quantitative yield over two steps) upon removal of the solvent.

$^{1}H$ NMR (500 MHz, DMSO-$d_6$) δ 8.61 (d, $J$ = 8.9 Hz, 1H), 8.60 (br, s, 2H), 8.27 (br, t, $J$ = 5.9 Hz, 1H), 6.57 (d, $J$ = 8.9 Hz, 1H), 4.21 (t, $J$ = 5.7 Hz, 1H), 4.08 – 3.89 (m, 2H). $^{13}C$ NMR (125 MHz, DMSO-$d_6$) δ 169.5, 152.5, 152.1, 148.2, 135.2, 129.7, 98.5, 51.7, 43.1. HRMS (m/z, ESI): calcd for $C_9H_{10}N_5O_4Se^+$ $[M+H]^+$: 331.9892, found 331.9895.

**(2*R*,3*R*,4*R*,5*S*,6*R*)-3-(ethyl(7-nitrobenzo[c][1,2,5]selenadiazol-4-yl)amino)-6(hydroxymethyl) tetrahydro-2*H*-pyran-2,4,5-triol (14)**

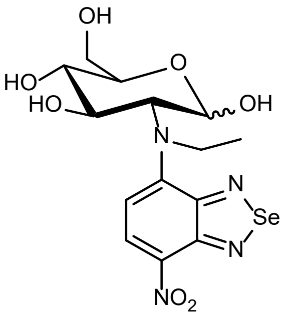

To a solution of 4-fluoro-7-nitrobenzo[c][1,2,5]selenadiazole **4F** (11 mg, 0.04 mmol, 1 eq) in MeCN (0.5 mL) was added 2-ethylamino-2-deoxy-D-glucose.[5] (9 mg, 0.04 mmol, 1 eq) in saturated $NaHCO_{3\,(aq)}$ (0.2 mL) and the reaction was stirred at r.t. for 48 h. The solvent was removed under reduced pressure and the crude product was purified by column chromatography (DCM:MeOH 9:1) to give compound **14** (3 mg, red solid, 16%, ~ 1.3:1 mixture of anomers).

$^1$H NMR (500 MHz, MeOD) δ 8.63 (d, *J* = 9.2 Hz, 1H), 8.62 (d, *J* = 9.2 Hz, 1H), 6.77 (d, *J* = 9.2 Hz, 1H), 6.66 (d, *J* = 9.4 Hz, 1H), 5.73 (d, *J* = 3.2 Hz, 1H), 5.59 – 5.52 (m, 1H), 5.42 (dd, *J* = 10.7, 3.3 Hz, 1H), 5.07 (d, *J* = 8.1 Hz, 1H), 4.19 (dd, *J* = 10.7, 8.4 Hz, 1H), 4.10 (dd, *J* = 14.6, 7.2 Hz, 1H), 4.01 – 3.85 (m, 6H), 3.79 – 3.72 (m, 2H), 3.49 – 3.42 (m, 2H), 3.38 – 3.35 (m, 2H), 1.37 (t, *J* = 7.0 Hz, 3H), 1.33 (t, *J* = 6.9 Hz, 3H). $^{13}$C NMR (125 MHz, MeOD) δ 154.0, 153.9, 153.8, 153.6, 150.9, 150.6, 133.2, 133.0, 129.9, 129.7, 105.4, 104.7, 94.5, 93.5, 76.5, 72.9, 72.3, 71.7, 70.6, 67.2, 65.4, 61.4, 40.5, 11.8, 11.1.

HRMS (m/z, ESI): calcd for $C_{14}H_{19}N_4O_7Se^+$ $[M+H]^+$: 435.0419, found 435.1001.

**(2*R*,3*R*,4*R*,5*S*,6*R*)-6-(hydroxymethyl)-3-((7-nitrobenzo[c][1,2,5]selenadiazol-4-yl)amino)tetrahydro-2*H*-pyran-2,4,5-triol (15)**

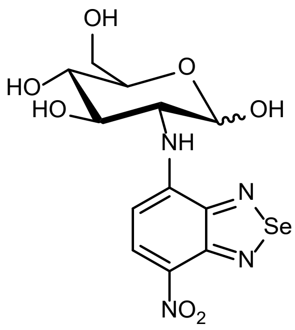


To a solution of 4-fluoro-7-nitrobenzo[c][1,2,5]selenadiazole **4F** (30 mg, 0.14 mmol, 1 eq) in MeCN (0.8 ml) was added 2-amino-2-deoxy-D-glucose hydrochloride (39 mg, 0.16 mmol, 1.1 eq) in saturated $NaHCO_{3\,(aq)}$ (0.8 mL) and the reaction was stirred at 30 $^{o}$C for 24 h. The solvent was removed under reduced pressure and the crude product was purified by column chromatography (DCM:MeOH 9:1) to give compound **15** (5 mg, orange solid, 13%, ~ 1.8:1 mixture of anomers ).

$^{1}$H NMR (500 MHz, DMSO-$d_6$) δ 8.55 (d, *J* = 7.4 Hz, 1H), 7.03 (s, 1H), 6.57 (d, *J* = 7.4 Hz, 1H), 5.17 (s, 2H), 5.10 – 5.03 (m, 1H), 4.52 – 4.45 (m, 1H), 3.85 – 3.65 (m, 4H), 3.50 – 3.47 (m, 1H). $^{13}$C NMR (125 MHz, DMSO-$d_6$) 152.1, 148.5, 135.6, 128.7, 99.0, 90.5, 73.3, 73.0, 70.8, 61.5, 58.2.

HRMS (m/z, ESI): calcd. for $C_{12}H_{15}N_4O_7Se^{+}$ $[M+H]^{+}$: 407.0092, found: 407.0100.

**6-oxo-6-(((3*R*,4*R*,5*S*,6*R*)-2,4,5-trihydroxy-6-(hydroxymethyl)tetrahydro-2*H*-pyran-3-yl)amino)hexanoic acid (pre-16)**

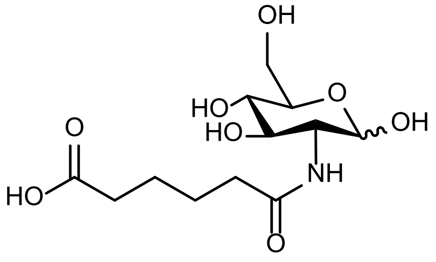

2-Amino-2-deoxy-D-glucose hydrochloride (216 mg, 1 mmol, 1 eq) was dissolved in DMF (5 mL) and $H_2O$ (1 mL). Oxepane-2,7-dione (128 mg, 1 mmol, 1 eq) was then added followed by *N*-methylmorpholine (0.2 mL) and reaction was stirred at r.t. overnight. The expected product **pre-16** was then precipitated with $Et_2O$ to give 252 mg of an off-white solid, that was used without further purification.

HRMS (m/z, ESI): calcd. for $C_{12}H_{21}NO_8Na$ $[M+Na]^+$: 330.1159, found: 330.1160.

**4-(2,2-bis(4-methoxyphenyl)-1-phenylvinyl)aniline (pre-17)[6]**

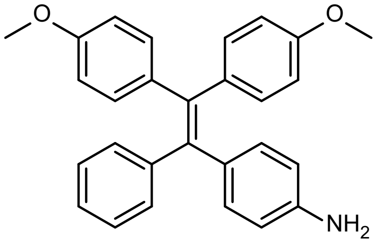

To a dry two-neck round bottom flask containing Zn powder (1.7 g, 26.0 mmol, 8 eq), were added pyridine (0.4 mL, 4.96 mmol, 1.5 eq) and anhydrous THF (30 mL) under a $N_2$ atmosphere. After cooling to 0 °C in an ice bath, $TiCl_4$ (2.0 mL, 18.2 mmol, 5.5 eq) was slowly injected and the mixture was heated under reflux for 30 min. A solution of 4,4'-dimethoxybenzophenone (801 mg, 4.1 mmol, 1.2 eq) and 4-aminobenzophenone (652 mg, 3.3 mmol, 1 eq) in anhydrous THF (20 mL) was then injected and the mixture was heated under reflux at 90 °C for 1 h. The reaction was then cooled down to r.t., quenched by slowly adding saturated $K_2CO_3$ solution (25 mL) and concentrated to dryness under reduced pressure. The crude product was purified by column chromatography (Hexane:EtOAc 8:2 → 7:3) to give the compound **pre-17** (222 mg, yellow solid,16%).

$^{1}$H NMR (500 MHz, $CDCl_3$) δ 7.07 – 7.14 (m, 3H), 7.02 – 7.05 (m, 2H), 7.01 – 6.88 (m, 6H), 6.74 – 6.61 (m, 6H), 3.78 (s, 3H), 3.75 (s, 3H). $^{13}$C NMR (125 MHz, $CDCl_3$) δ 158.02, 157.97, 144.3, 139.5, 138.6, 136.51, 136.50, 132.61, 132.57, 132.54, 131.44, 131.37, 127.7, 127.2, 126.1, 117.1, 113.3, 113.1, 113.0, 55.09, 55.08.

HRMS (m/z, ESI): calcd. for $C_{28}H_{26}NO_2$ $[M+H]^+$: 408.1958, found: 408.1937.

**_N_-ethyl-_N_-(5-(6-oxo-6-(((3_R_,4_R_,5_S_,6_R_)-2,4,5-trihydroxy-6-(hydroxymethyl) tetrahydro-2_H_-pyran-3-yl)amino)hexanamido)-9_H_-benzo[a]phenoxazin-9-ylidene)ethanaminium (16)**

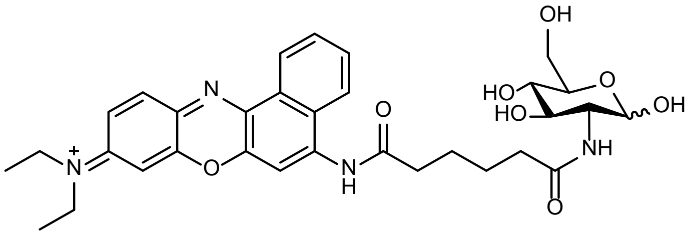


6-Oxo-6-(((3*R*,4*R*,5*S*,6*R*)-2,4,5-trihydroxy-6-(hydroxymethyl)tetrahydro-2*H*-pyran-3-yl) amino)hexanoic acid **pre-16** (10 mg, 0.03 mmol, 1 eq) was dissolved in DMF (0.5 mL), then COMU (21 mg, 0.05 mmol, 1.5 eq) was added and the mixture was stirred at r.t. for 5 min. Nile Blue (13 mg, 0.03 mmol, 1 eq) and *N,N*-diisopropylethylamine (18 μL, 0.1 mmol, 3 eq) were then added and reaction was stirred at r.t. for 2 h. It was then purified by semi-preparative HPLC to give compound **16** (6 mg, blue solid, 30%, mixture of anomers).

$^{1}$H NMR (500 MHz, DMSO-$d_6$, main anomer signals) δ 8.14 (s, 1H), 7.76 – 7.65 (m, 2H), 7.63 – 7.48 (m, 2H), 6.90 – 6.75 (m, 1H), 6.50 – 6.46 (m, 1H), 6.45 – 6.36 (m,1H), 5.01 – 4.85 (m, 3H), 4.64 – 4.36 (m, 3H), 3.73 – 3.57 (m, 5H), 3.18 – 3.03 (m, 2H), 2.45 – 2.32 (m, 1H), 2.22 – 2.10 (3H), 1.75 – 1.54 (m, 4H), 1.29 – 1.09 (m, 6H). $^{13}$C NMR (125 MHz, DMSO-$d_{6,}$ main anomer signals) δ 174.9, 172.7, 172.5, 169.3, 163.5, 159.8, 153.0, 149.3, 147.3, 136.4, 131.8, 130.5, 129.4, 126.2, 124.2, 122.7, 120.3, 119.0, 96.0, 91.1, 77.3, 74.8, 72.5, 71.6, 71.4, 71.0, 61.6, 57.6, 54.7, 44.8, 36.5, 36.0, 34.0, 31.2, 25.6, 24.8, 12.7. HRMS (m/z, ESI): calcd. for $C_{32}H_{39}N_4O_8$ [M]$^+$: 607.2762, found: 607.2754.

***N*'-1-(4-(2,2-bis(4-methoxyphenyl)-1-phenylvinyl)phenyl)-*N*'-6-((3*R*,4*R*,5*S*,6*R*)-2,4,5-trihydroxy-6-(hydroxymethyl)tetrahydro-2*H*-pyran-3-yl)adipamide (17)**

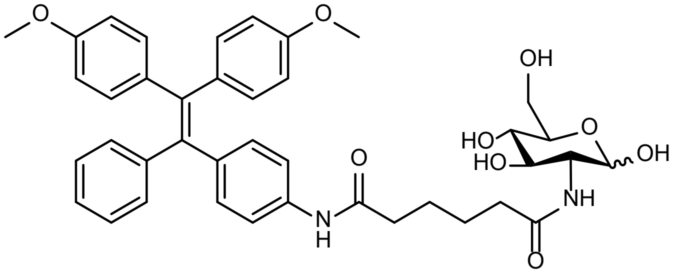


6-Oxo-6-(((3*R*,4*R*,5*S*,6*R*)-2,4,5-trihydroxy-6-(hydroxymethyl)tetrahydro-2*H*-pyran-3-yl) amino)hexanoic acid **pre-16** (20 mg, 0.06 mmol, 1 eq) was dissolved in DMF (1 mL), then COMU (43 mg, 0.1 mmol, 1.5 eq) was added and the mixture was stirred at r.t. for 5 min. 4-(2,2-Bis(4-methoxyphenyl)-1-phenylvinyl)aniline **pre-17** (27 mg, 0.06 mmol, 1 eq) and *N,N*-diisopropylethylamine (36 μL, 0.2 mmol, 3 eq) were then added and reaction was stirred at r.t. for 3 h. It was then purified by semi-preparative HPLC to give compound **17** (7 mg, off-white solid, 15%, one major anomer containing traces of a minor anomer).

$^1$H NMR (500 MHz, DMSO-$d_6$) δ 9.83 (s, 1H), 8.46 (br, s, 1H), 7.35 (d, *J* = 8.5 Hz, 2H), 7.22 – 7.04 (m, 3H), 7.02 – 6.92 (m, 2H), 6.90 – 6.83 (m, 6H), 6.72 (d, *J* = 8.8 Hz, 2H), 6.68 (d, *J* = 8.8 Hz, 2H), 4.93 (s, 2H), 4.70 – 4.48 (m, 1H), 4.46 – 4.37 (m, 1H), 3.69 (s, 3H), 3.67 (s, 3H), 3.63 – 3.56 (m, 2H), 3.54 – 3.44 (m, 2H), 3.23 – 2.99 (m, 2H), 2.26 (t, *J* = 6.9 Hz, 2H), 2.18 – 2.08 (m, 2H), 1.61 – 1.46 (m, 4H). $^{13}$C NMR (125 MHz, DMSO-$d_6$) δ 173.0, 172.6, 171.6, 158.12, 158.09, 144.4, 139.8, 138.92, 138.86, 137.9, 136.34, 136.28, 132.5, 131.5, 131.2, 128.3, 126.7, 118.8, 113.7, 113.6, 96.1, 91.1, 77.3, 74.8, 72.5, 71.6, 71.3, 70.9, 61.6, 57.6, 55.39, 55.37, 54.8, 36.7, 36.0, 35.5, 25.4.

HRMS (m/z, ESI): calcd. for $C_{40}H_{45}N_2O_9$ [M+H]$^+$: 697.3120, found: 697.3139.

# $^1$H and $^{13}$C NMR spectra

## Compound **2a**

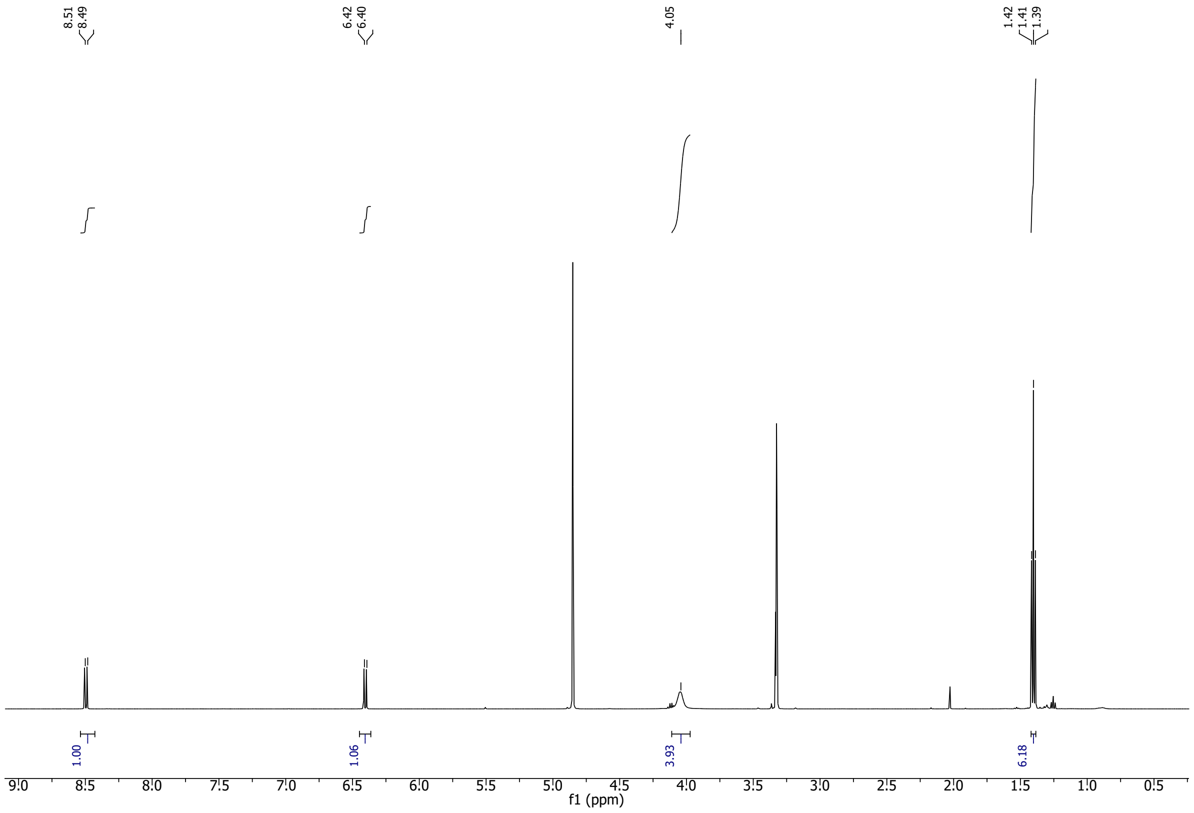


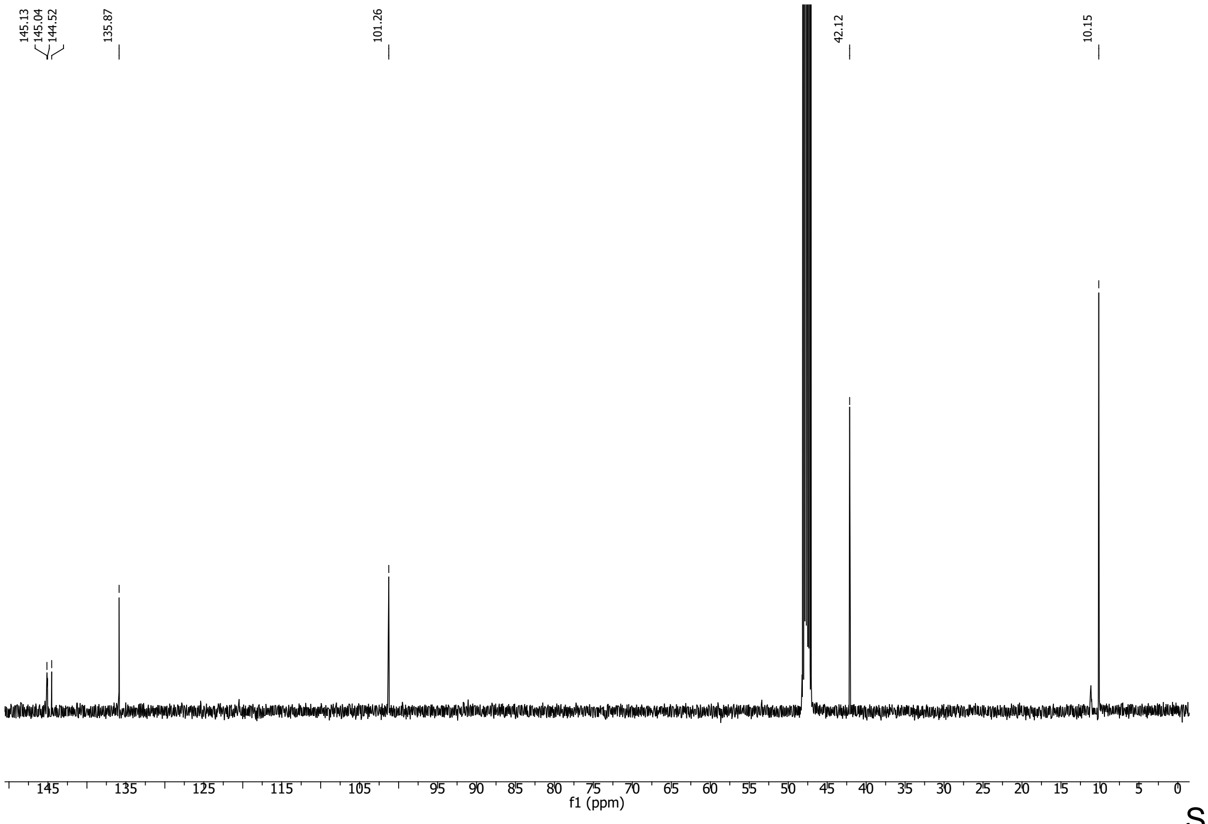

Compound **3a**

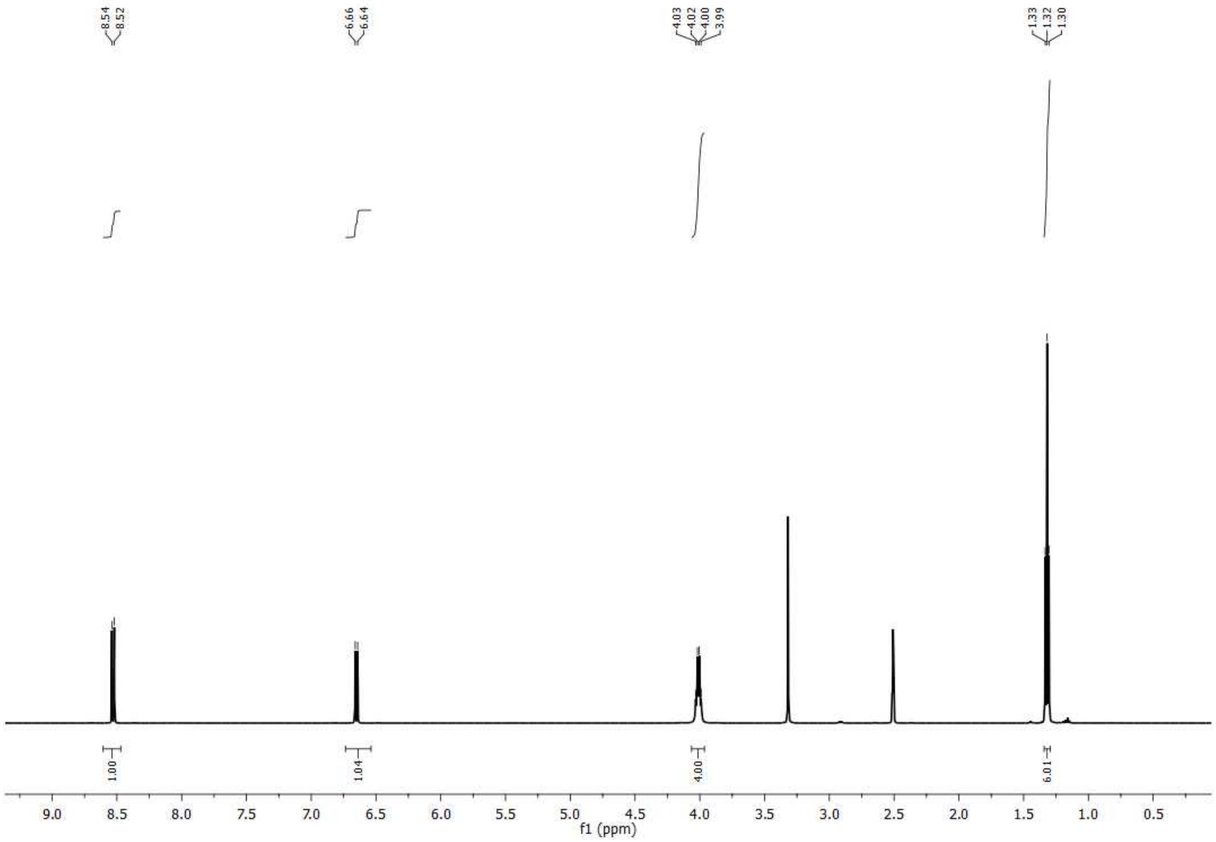
8.54
8.52
6.66
6.64
4.03
4.02
4.00
3.99
1.33
1.32
1.30
1.00
1.04
4.00
6.01
9.0
8.5
8.0
7.5
7.0
6.5
6.0
5.5
5.0
4.5
4.0
3.5
3.0
2.5
2.0
1.5
1.0
0.5
f1 (ppm)

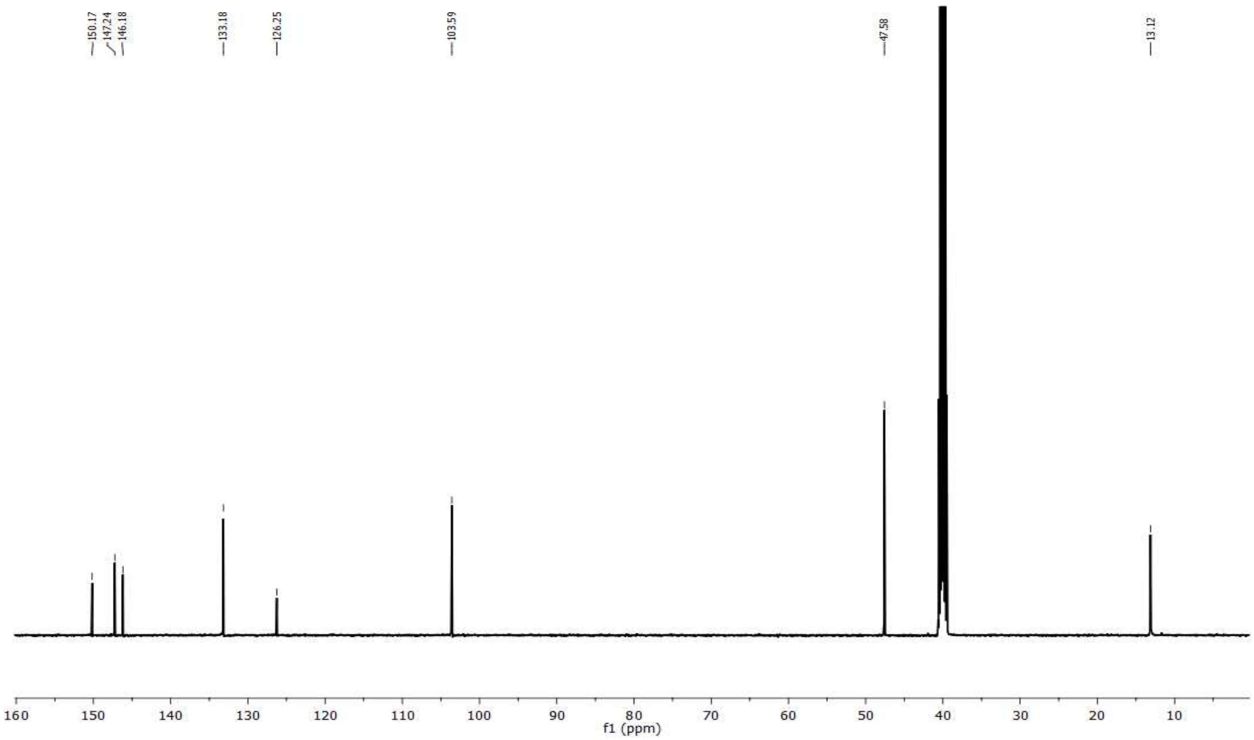
150.17
147.24
146.18
133.18
126.25
103.59
47.58
13.12
160
150
140
130
120
110
100
90
80
70
60
50
40
30
20
10
f1 (ppm)

Compound **4a**

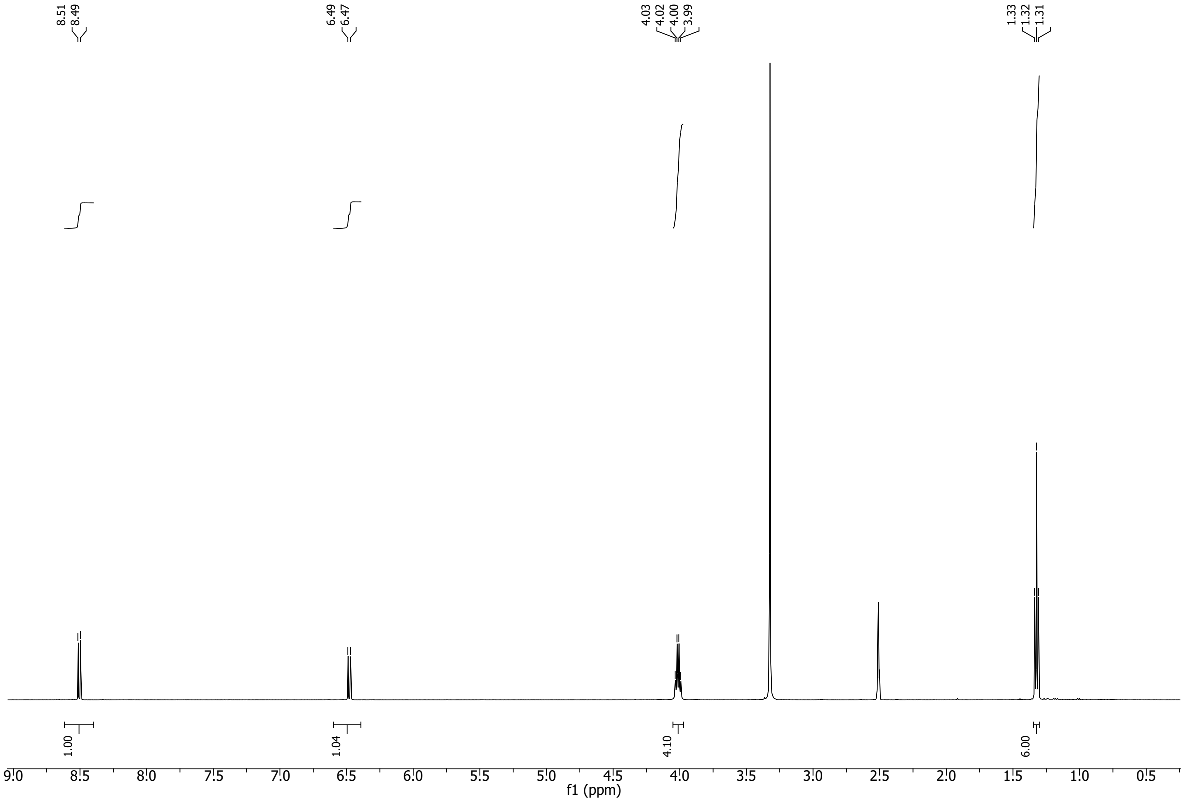
8.51
8.49
6.49
6.47
4.03
4.02
4.00
3.99
1.33
1.32
1.31
1.00
1.04
4.10
6.00
9.0 8.5 8.0 7.5 7.0 6.5 6.0 5.5 5.0 4.5 4.0 3.5 3.0 2.5 2.0 1.5 1.0 0.5
f1 (ppm)

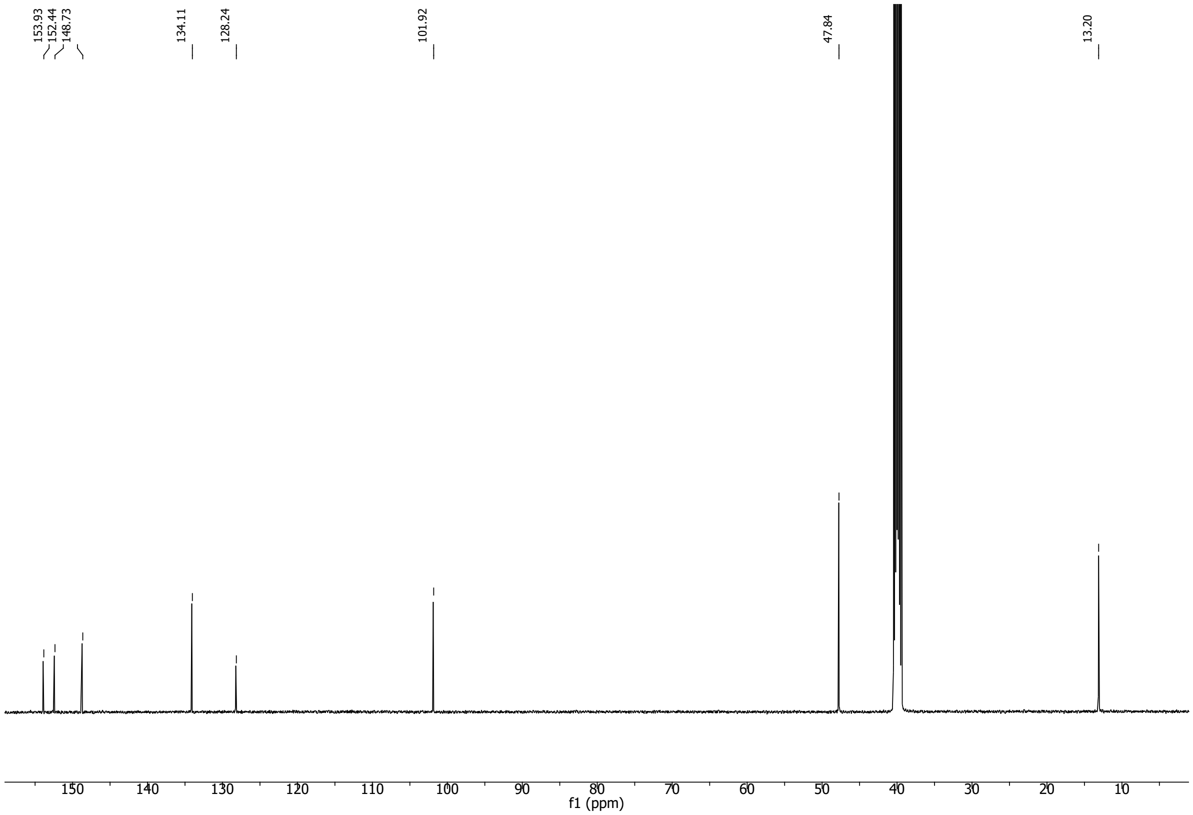
153.93
152.44
148.73
134.11
128.24
101.92
47.84
13.20
150 140 130 120 110 100 90 80 70 60 50 40 30 20 10
f1 (ppm)

Compound **5a**

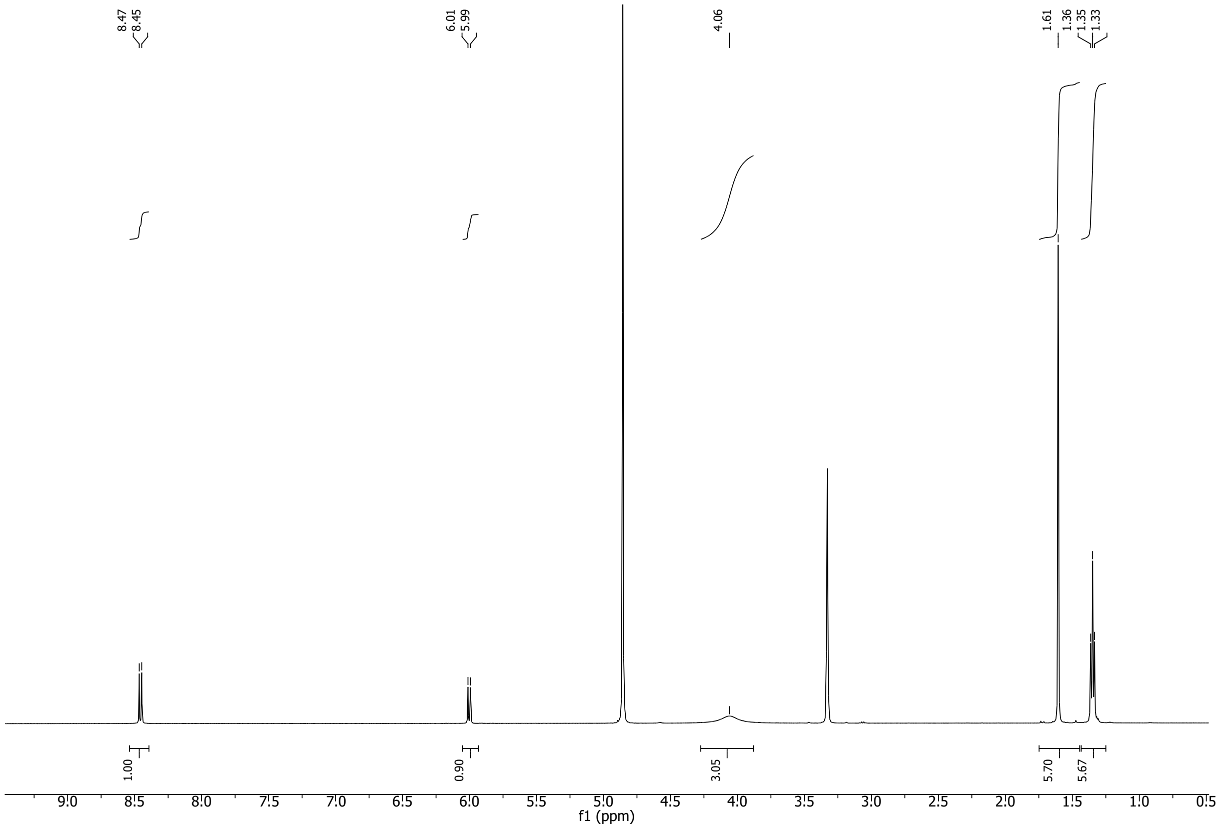
8.47
8.45
6.01
5.99
4.06
1.61
1.36
1.35
1.33
1.00
0.90
3.05
5.70
5.67
9.0 8.5 8.0 7.5 7.0 6.5 6.0 5.5 5.0 4.5 4.0 3.5 3.0 2.5 2.0 1.5 1.0 0.5
f1 (ppm)

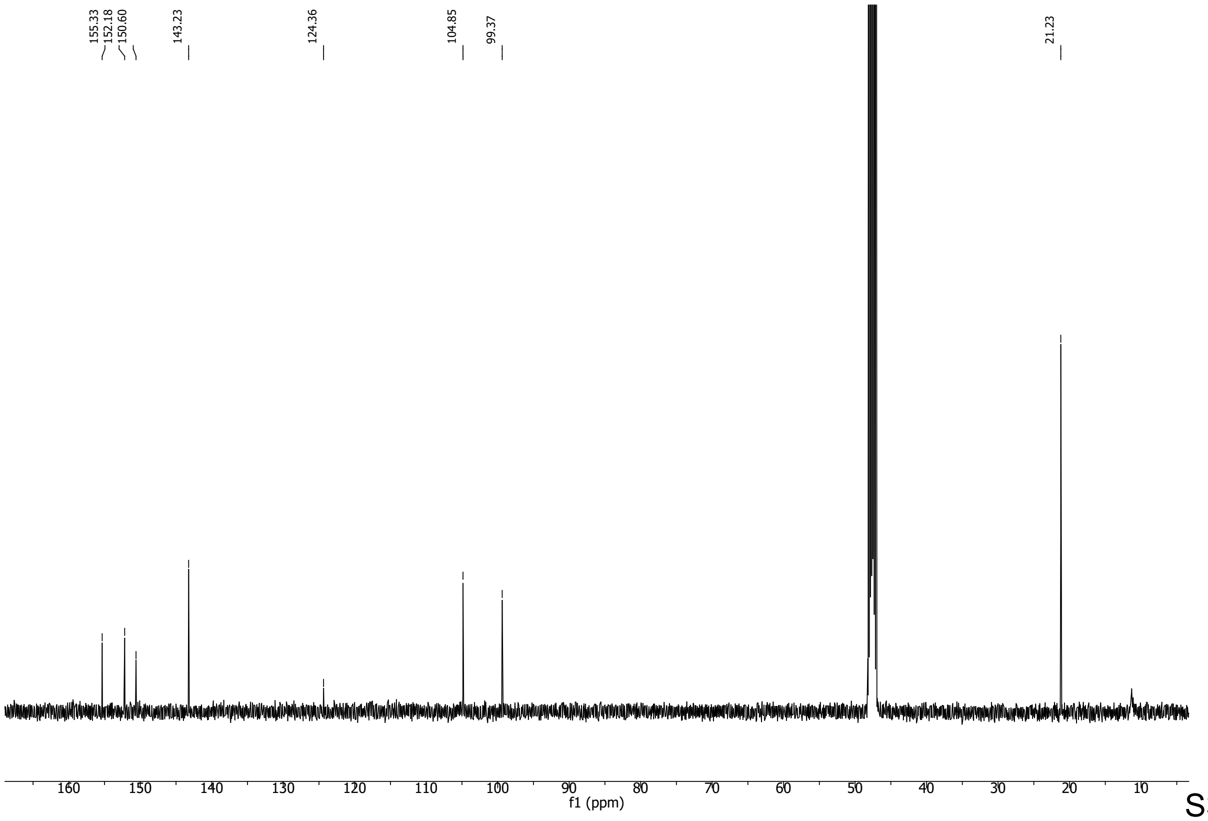
155.33
152.18
150.60
143.23
124.36
104.85
99.37
21.23
160 150 140 130 120 110 100 90 80 70 60 50 40 30 20 10
f1 (ppm)

Compound **6** (contains traces of residual DCM)

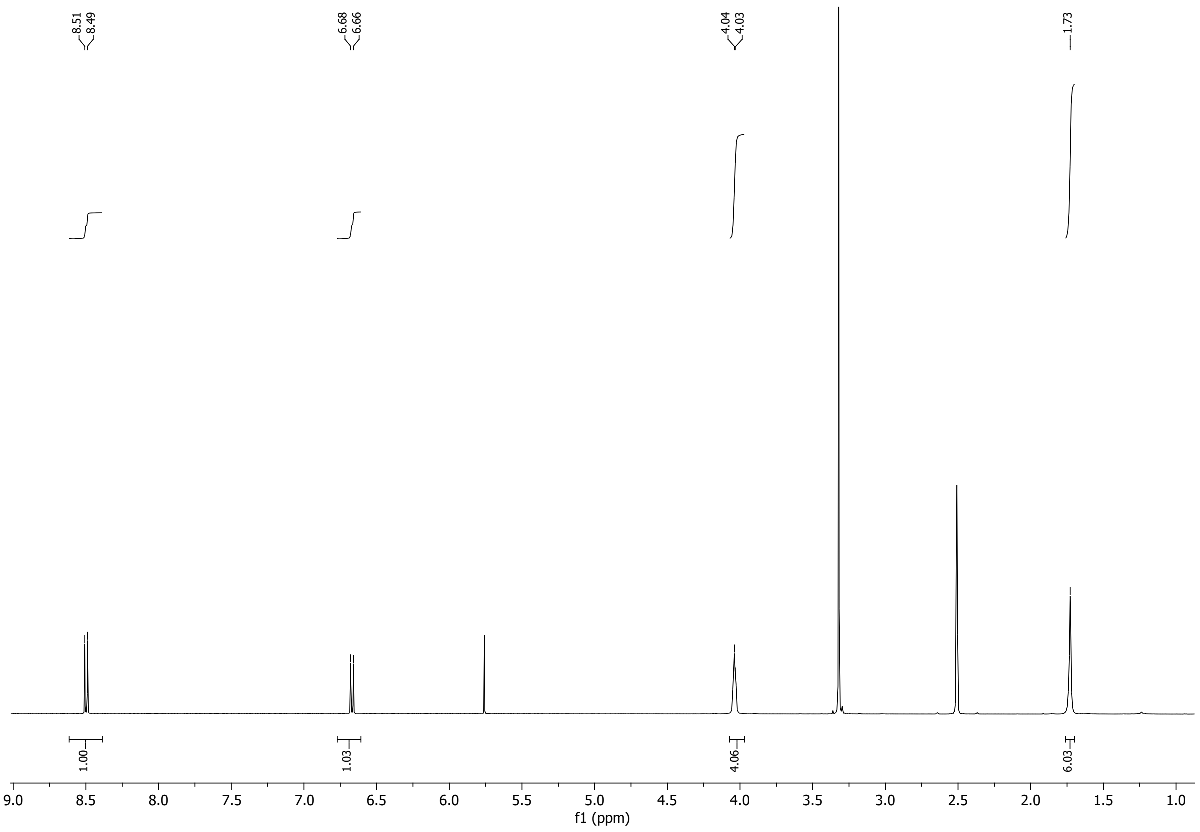


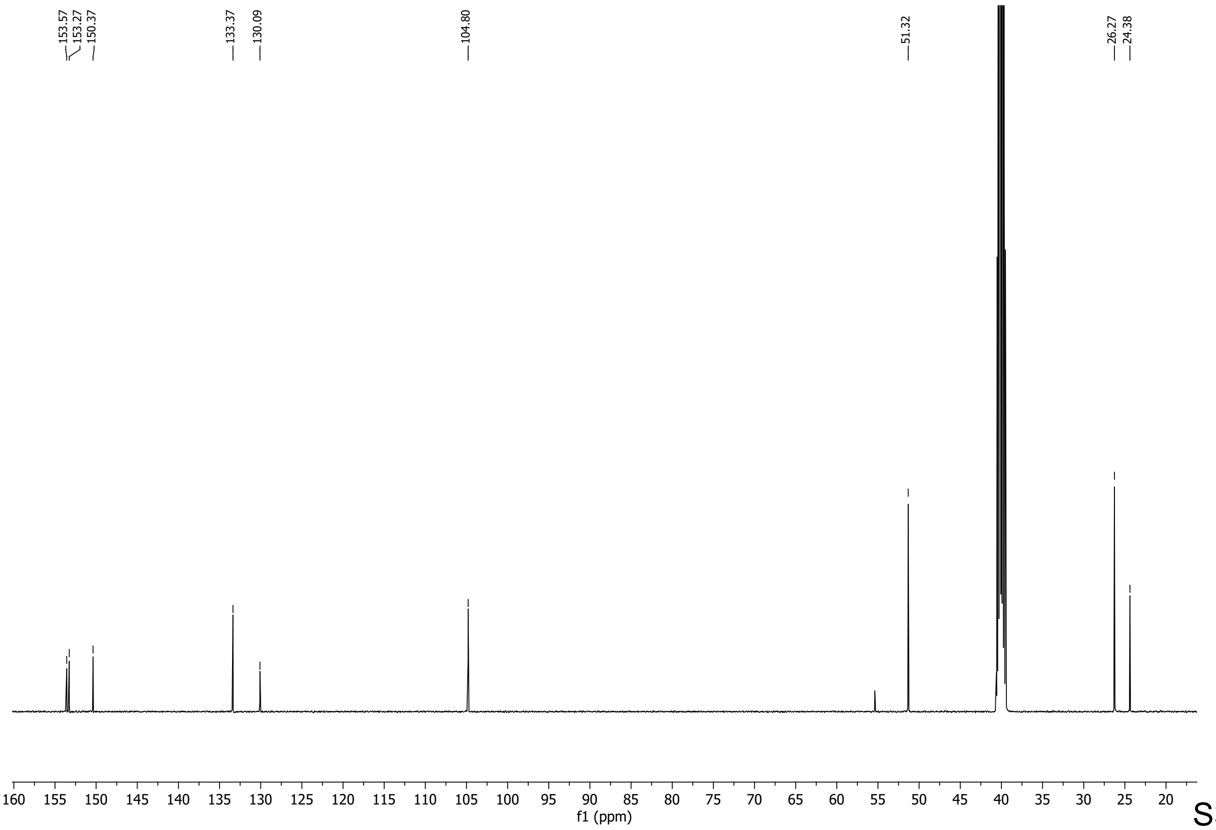

Compound **7** (contains traces of residual DCM)

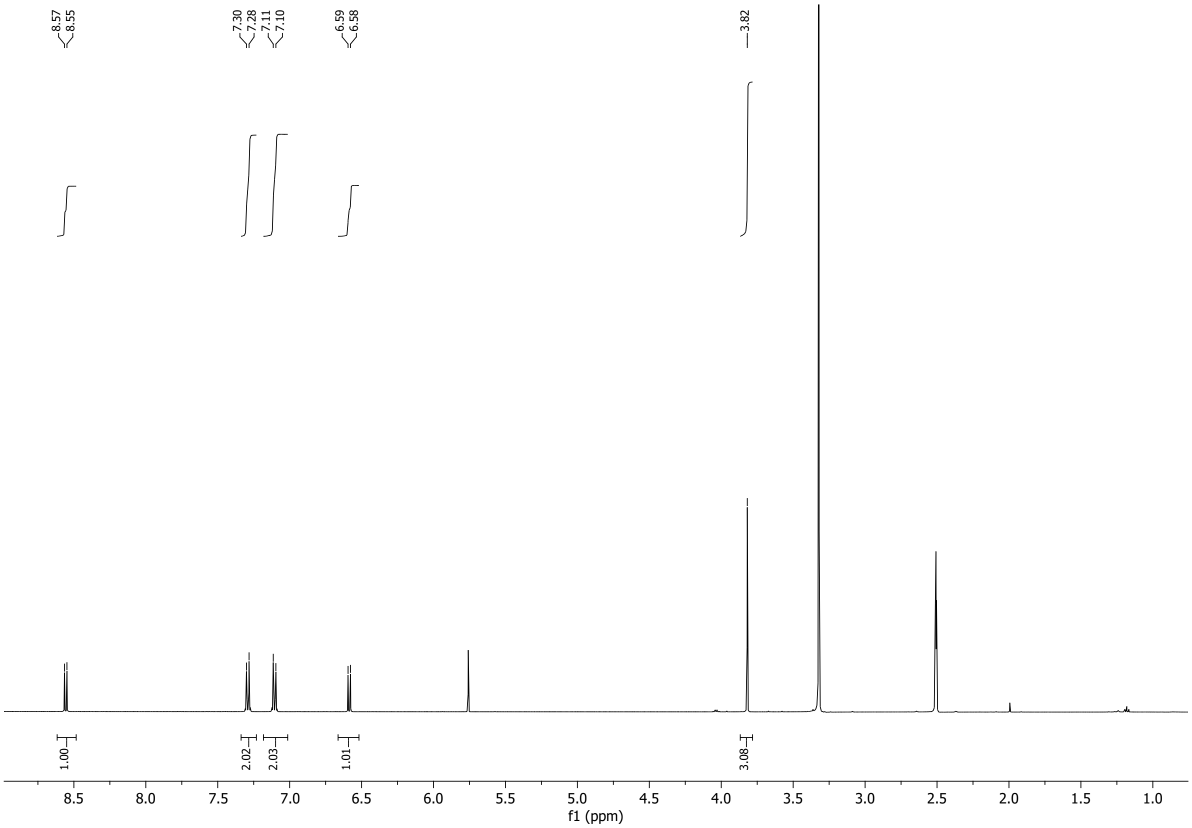


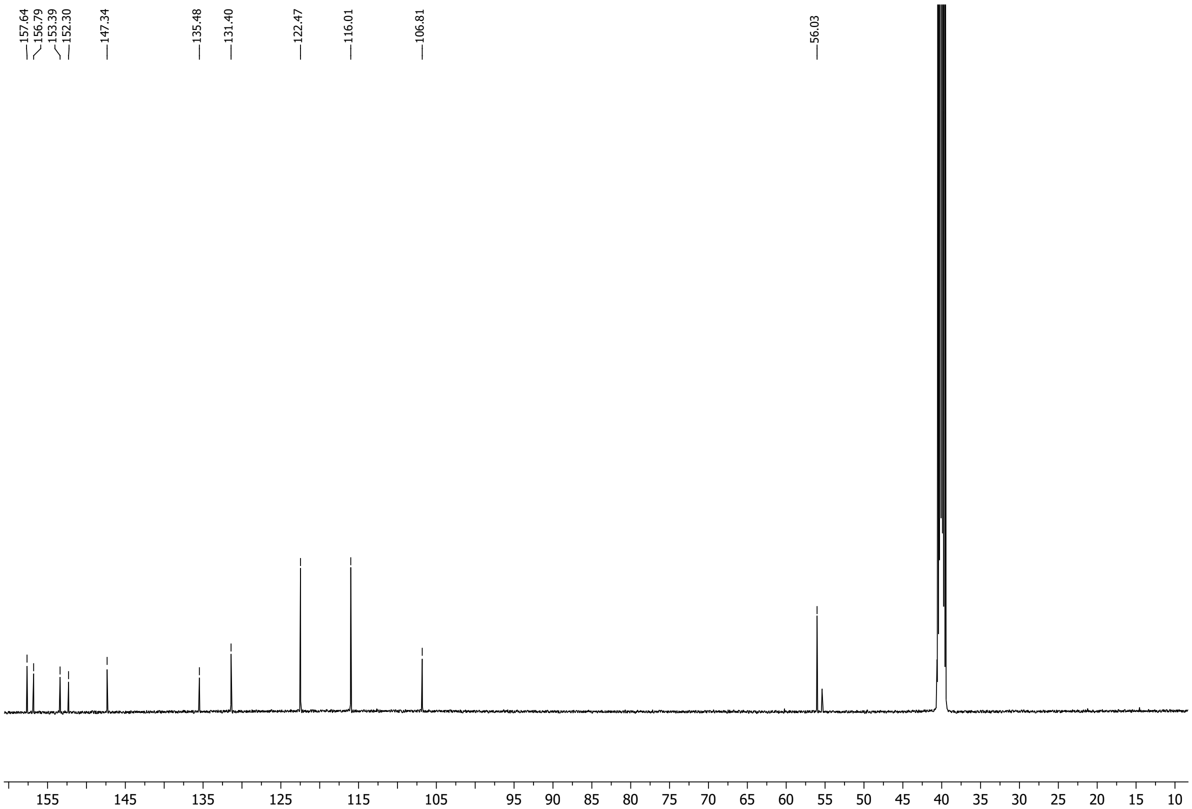

Compound **8**

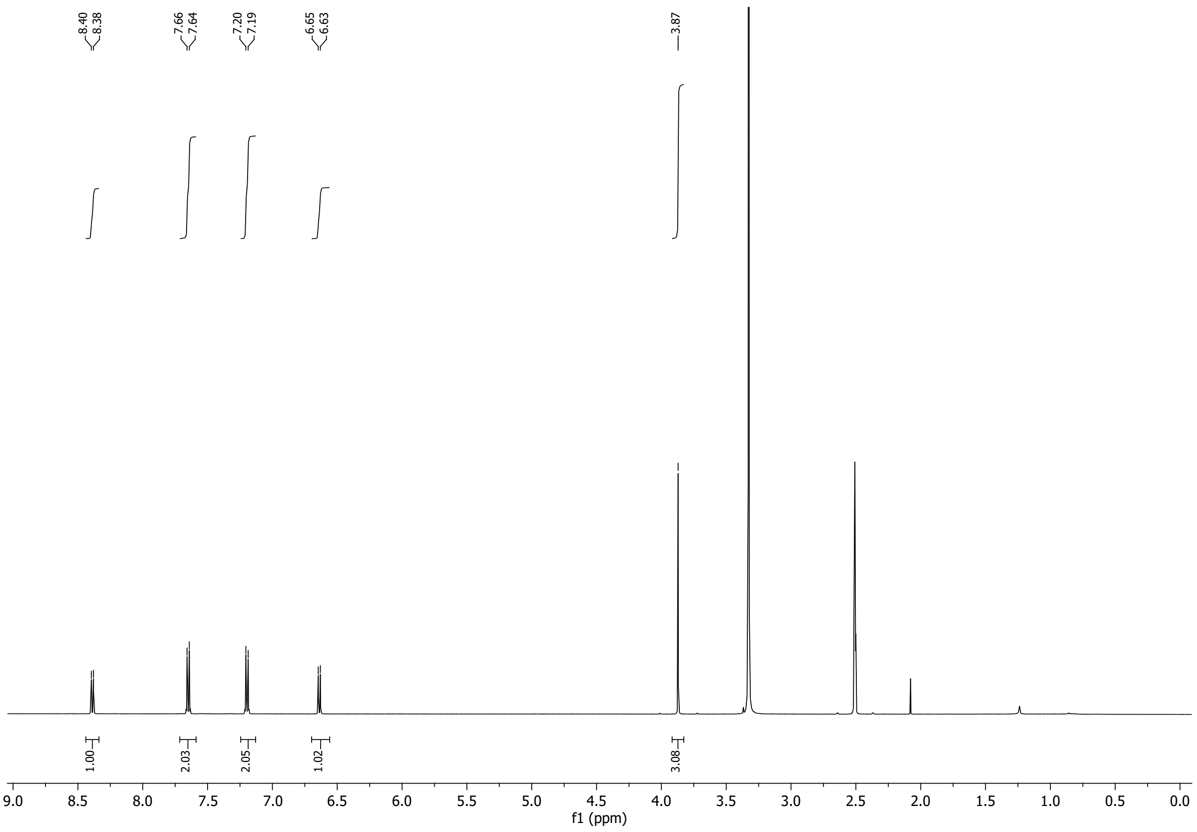
8.40
8.38
7.66
7.64
7.20
7.19
6.65
6.63
3.87
1.00
2.03
2.05
1.02
3.08
9.0 8.5 8.0 7.5 7.0 6.5 6.0 5.5 5.0 4.5 4.0 3.5 3.0 2.5 2.0 1.5 1.0 0.5 0.0
f1 (ppm)

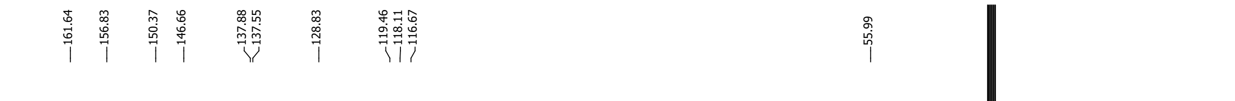
161.64
156.83
150.37
146.66
137.88
137.55
128.83
119.46
118.11
116.67
55.99
170 160 150 140 130 120 110 100 90 80 70 60 50 40 30 20 10
f1 (ppm)

Compound **9**

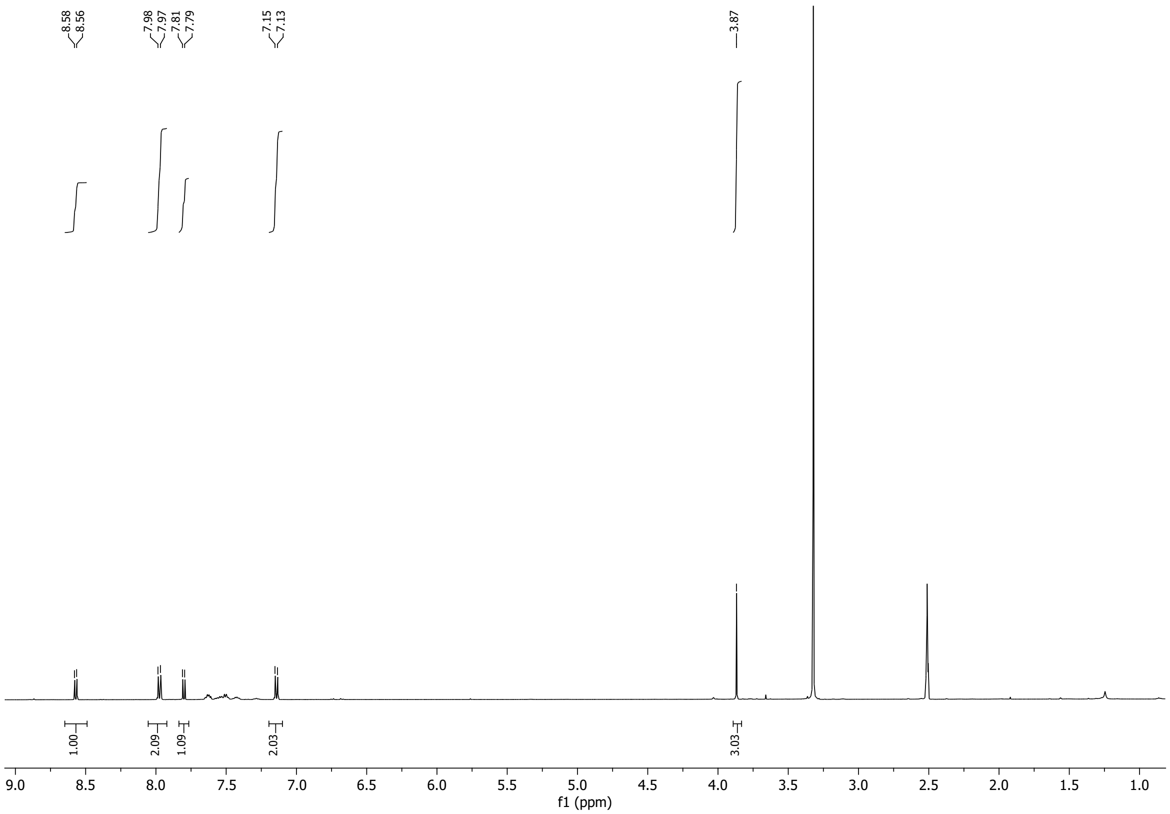
8.58
8.56
7.98
7.97
7.81
7.79
7.15
7.13
3.87
1.00
2.09
1.09
2.03
3.03
9.0 8.5 8.0 7.5 7.0 6.5 6.0 5.5 5.0 4.5 4.0 3.5 3.0 2.5 2.0 1.5 1.0
f1 (ppm)

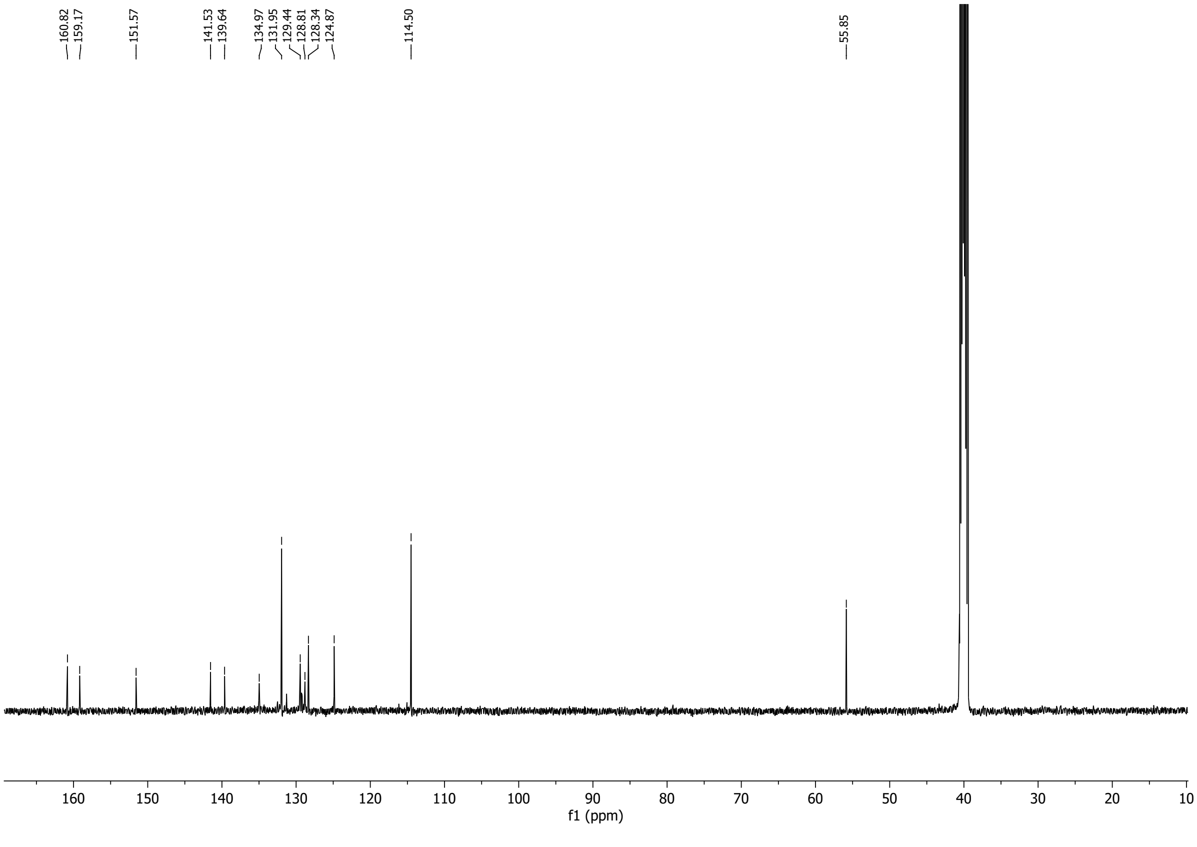
160.82
159.17
151.57
141.53
139.64
134.97
131.95
129.44
128.81
128.34
124.87
114.50
55.85
160 150 140 130 120 110 100 90 80 70 60 50 40 30 20 10
f1 (ppm)

Compound **11** (~65% purity with remaining non-nitrated intermediate)

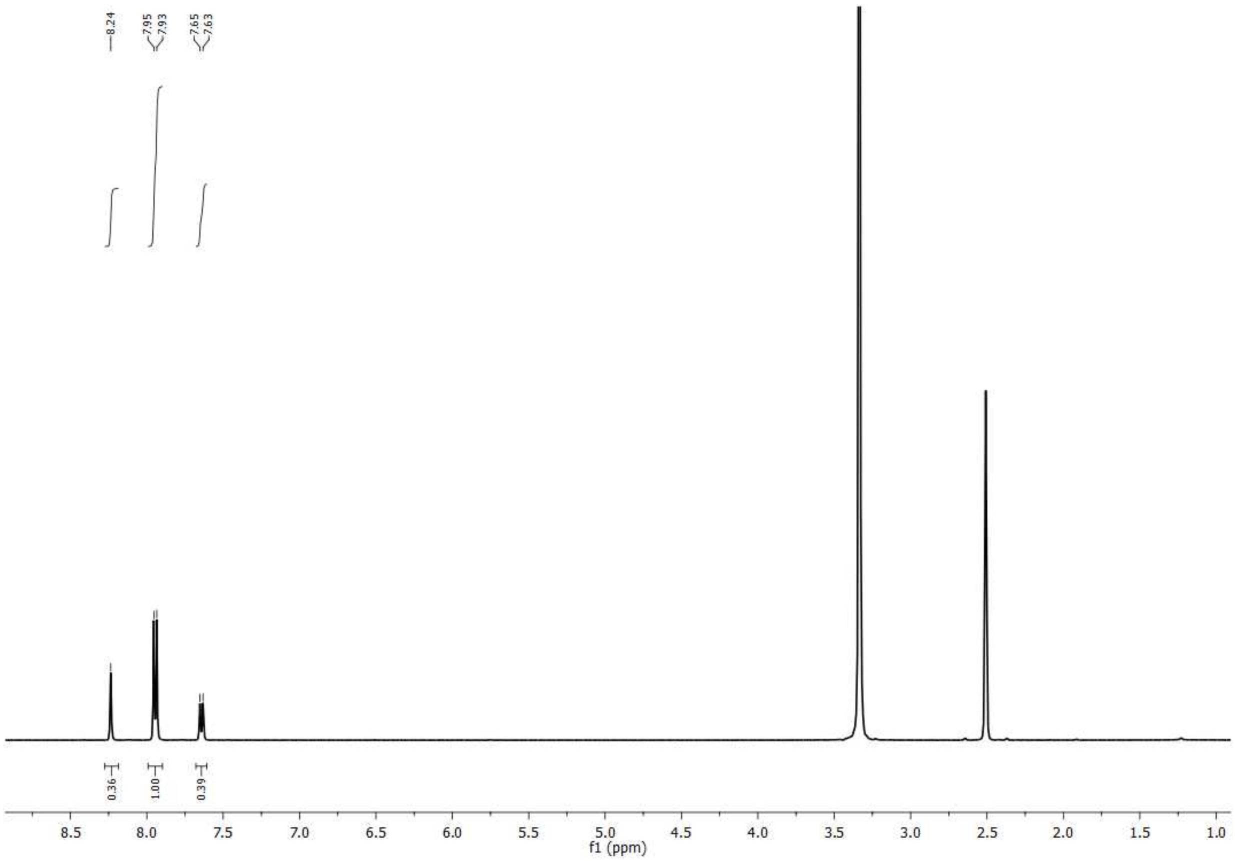

Compound **11a**

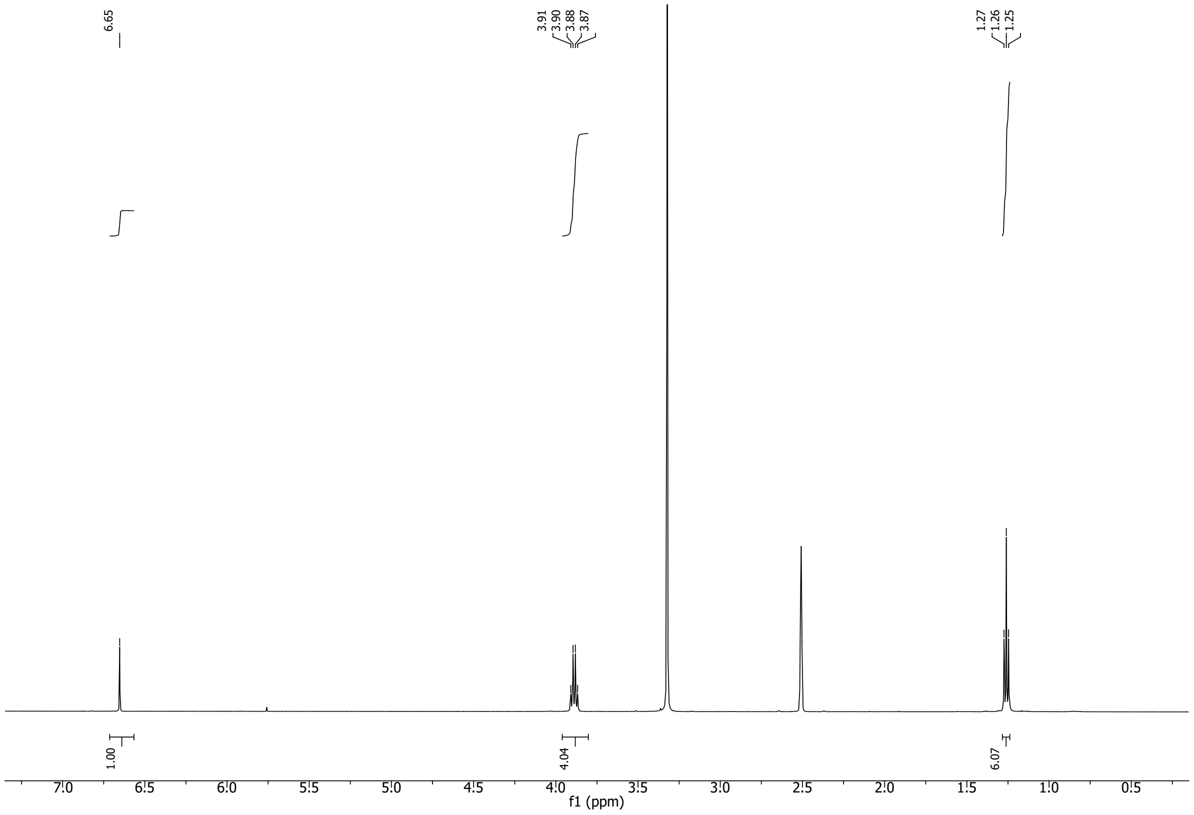


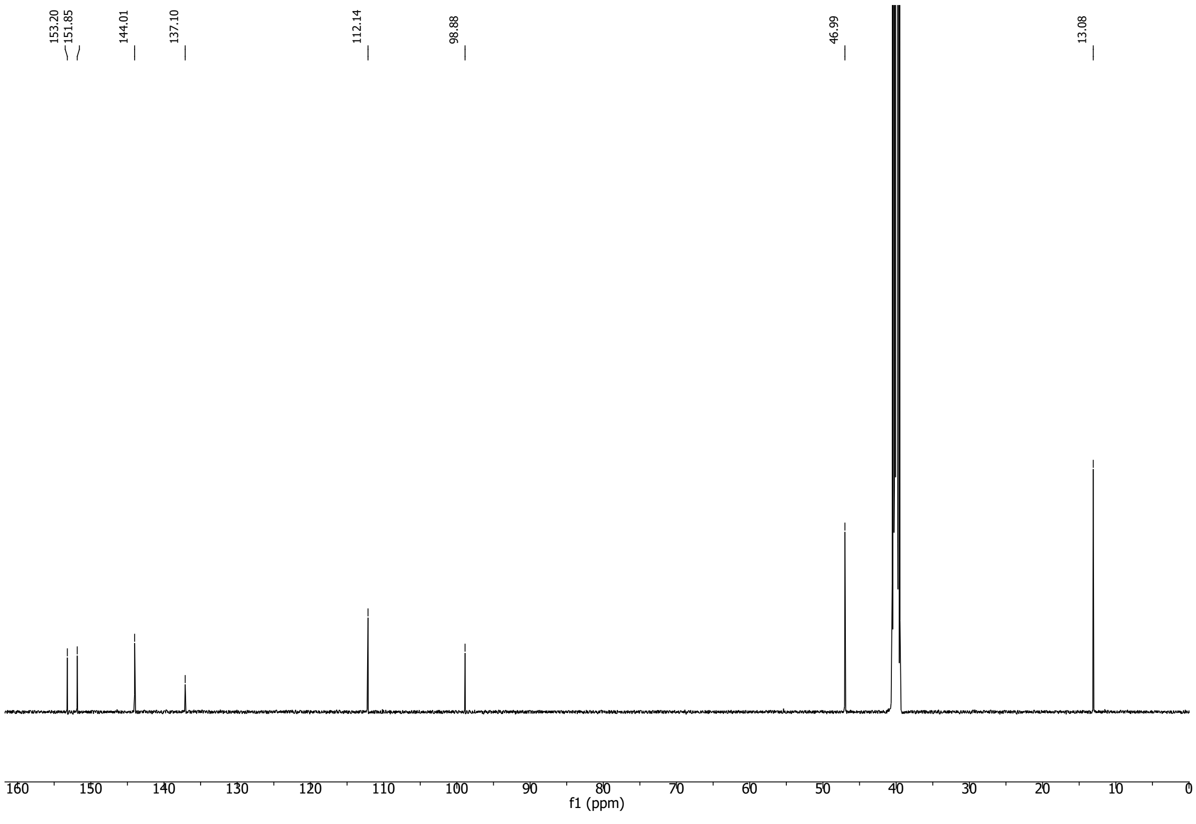

Compound **12**

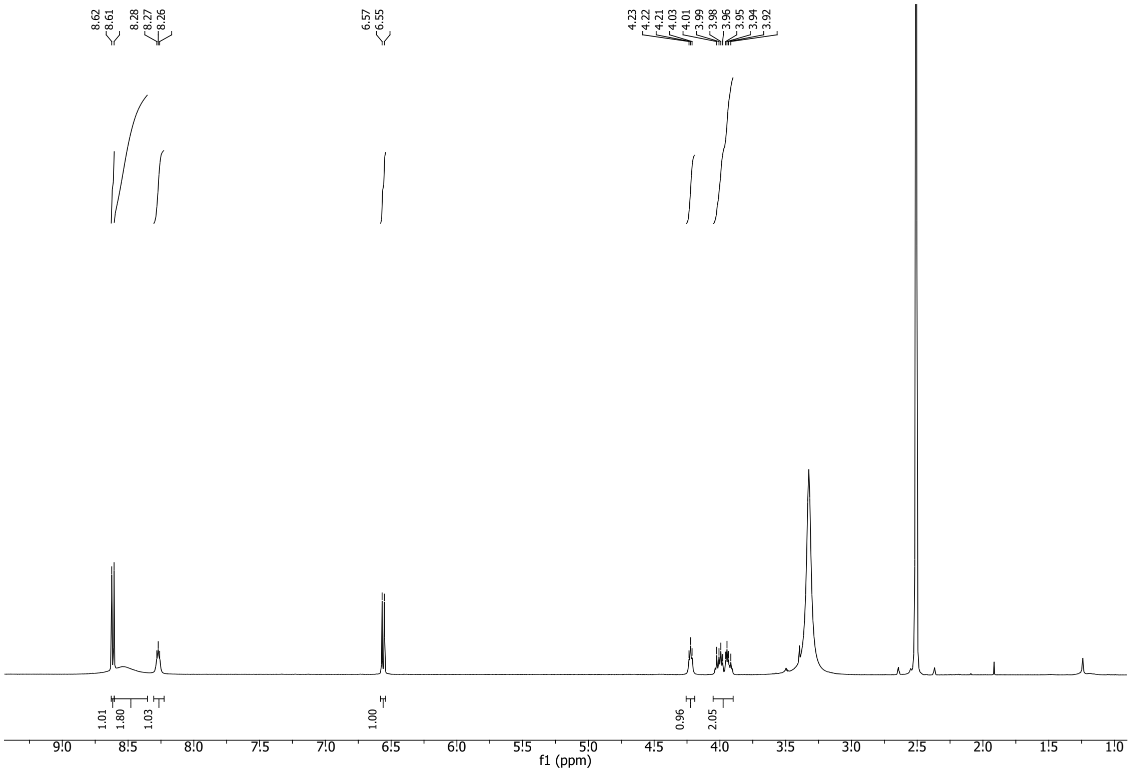
8.62
8.61
8.28
8.27
8.26
6.57
6.55
4.23
4.22
4.21
4.03
4.01
3.99
3.98
3.96
3.95
3.94
3.92
1.01
1.80
1.03
1.00
0.96
2.05
9.0 8.5 8.0 7.5 7.0 6.5 6.0 5.5 5.0 4.5 4.0 3.5 3.0 2.5 2.0 1.5 1.0
f1 (ppm)

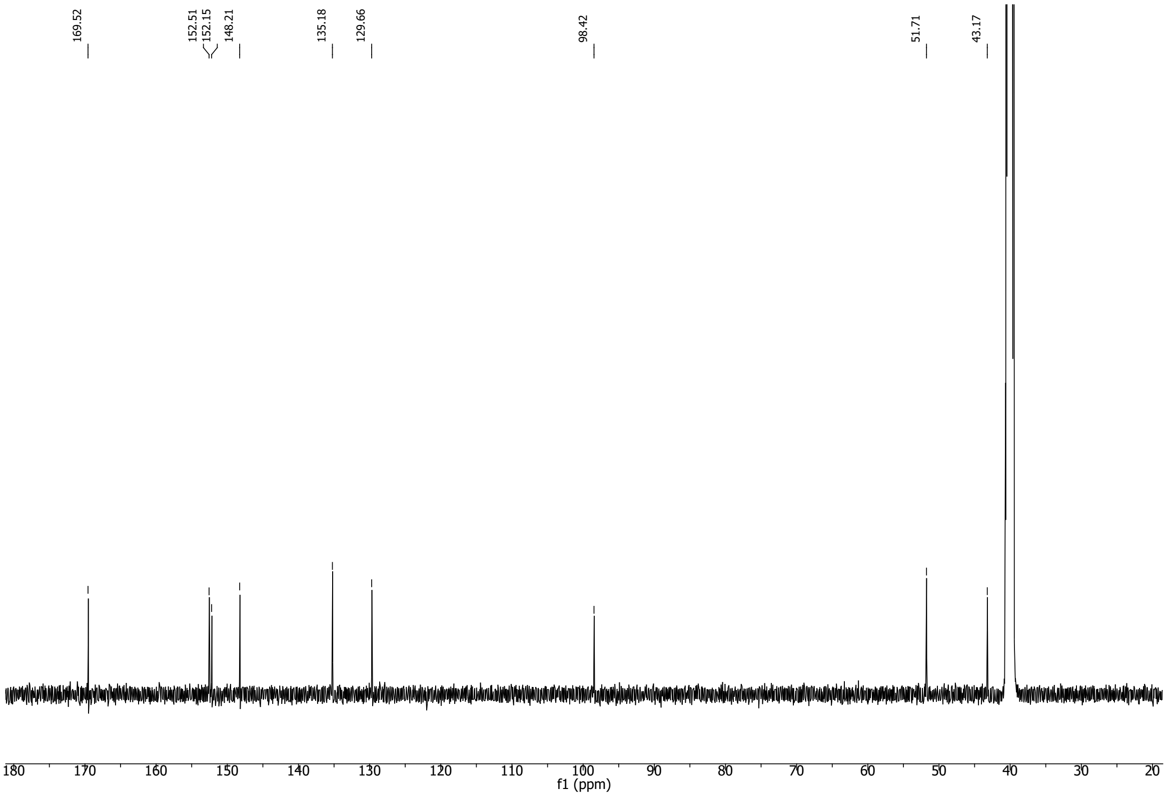
169.52
152.51
152.15
148.21
135.18
129.66
98.42
51.71
43.17
180 170 160 150 140 130 120 110 100 90 80 70 60 50 40 30 20
f1 (ppm)

Compound **13**

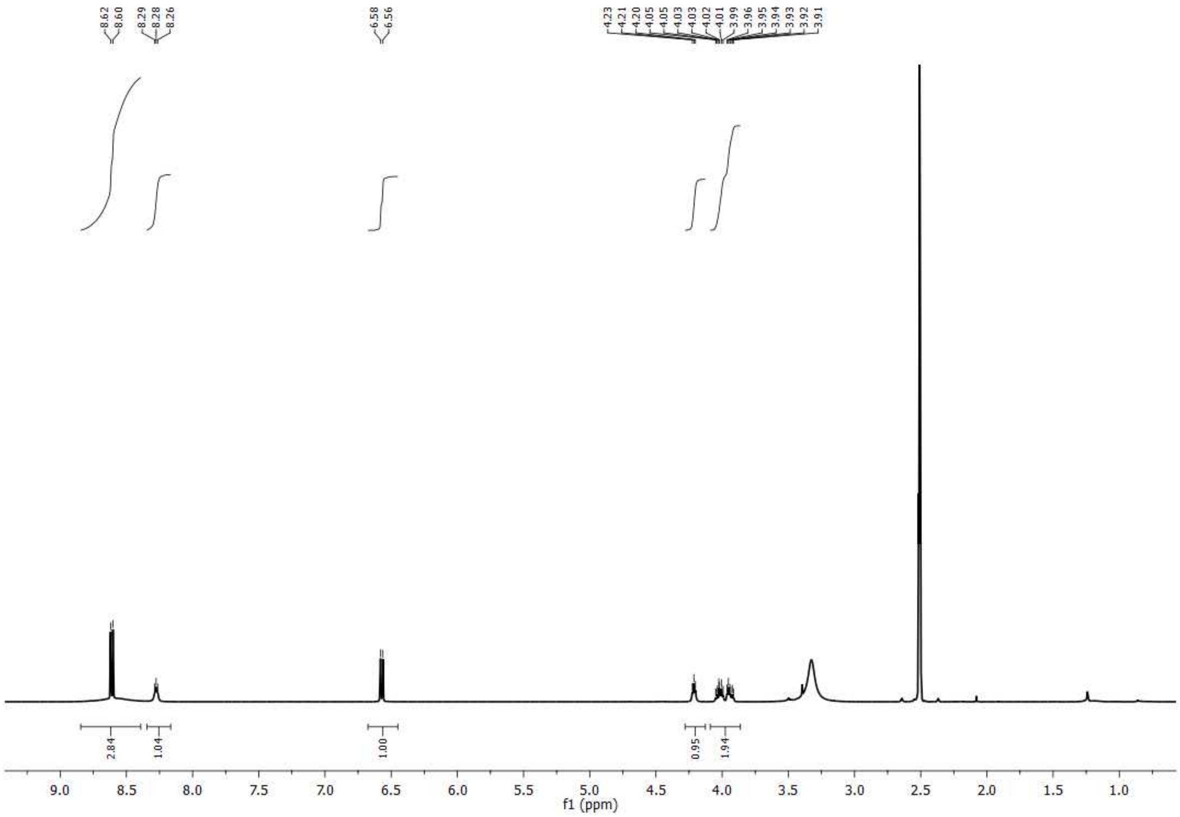
8.62
8.60
8.29
8.28
8.26
6.58
6.56
4.23
4.21
4.20
4.05
4.05
4.03
4.03
4.02
4.01
3.99
3.96
3.95
3.94
3.93
3.92
3.91
2.84
1.04
1.00
0.95
1.94
9.0
8.5
8.0
7.5
7.0
6.5
6.0
5.5
5.0
4.5
4.0
3.5
3.0
2.5
2.0
1.5
1.0
f1 (ppm)

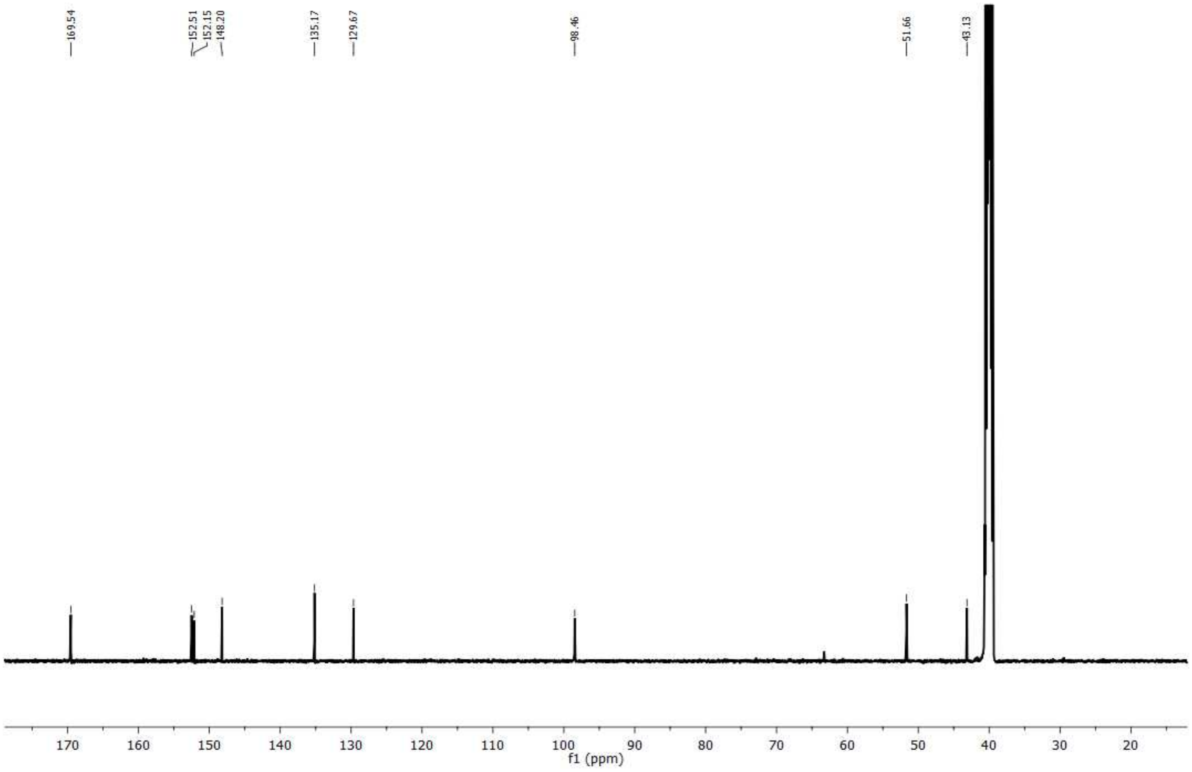
169.54
152.51
152.15
148.20
135.17
129.67
98.46
51.66
43.13
170
160
150
140
130
120
110
100
90
80
70
60
50
40
30
20
f1 (ppm)

Compound **14**

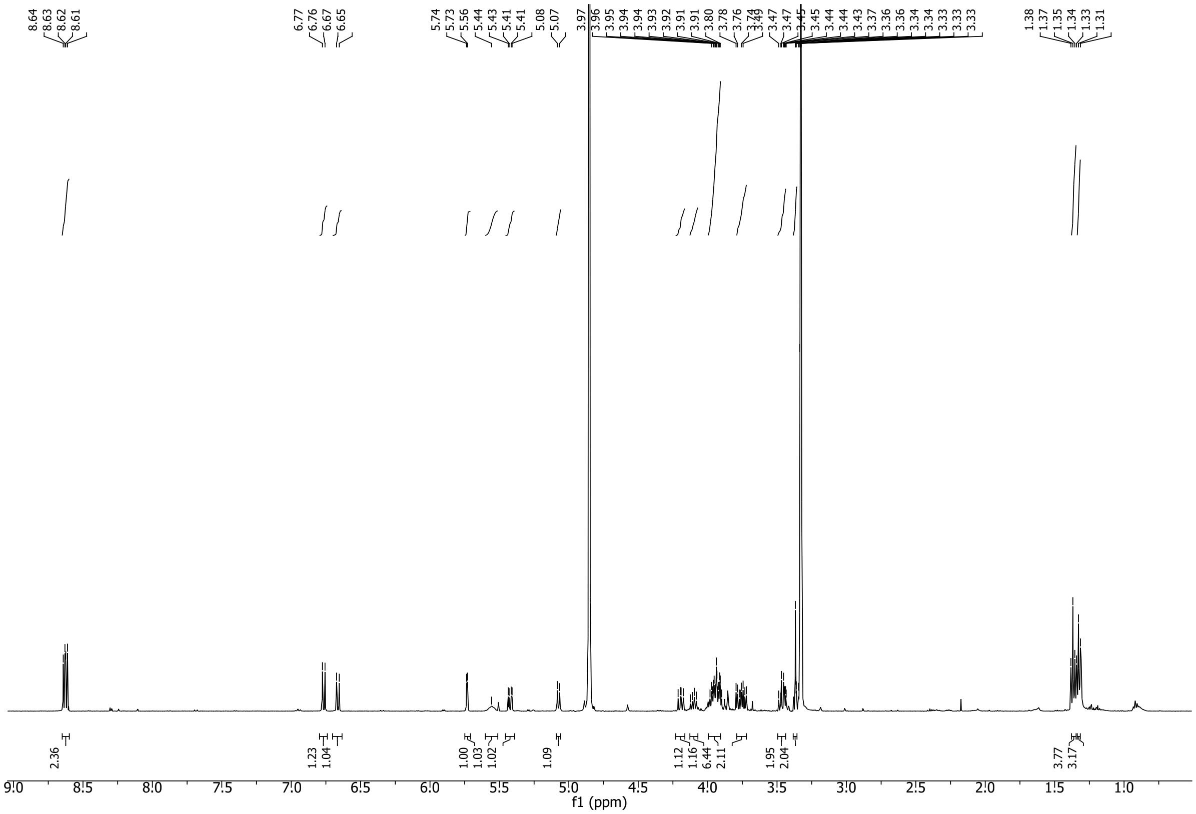

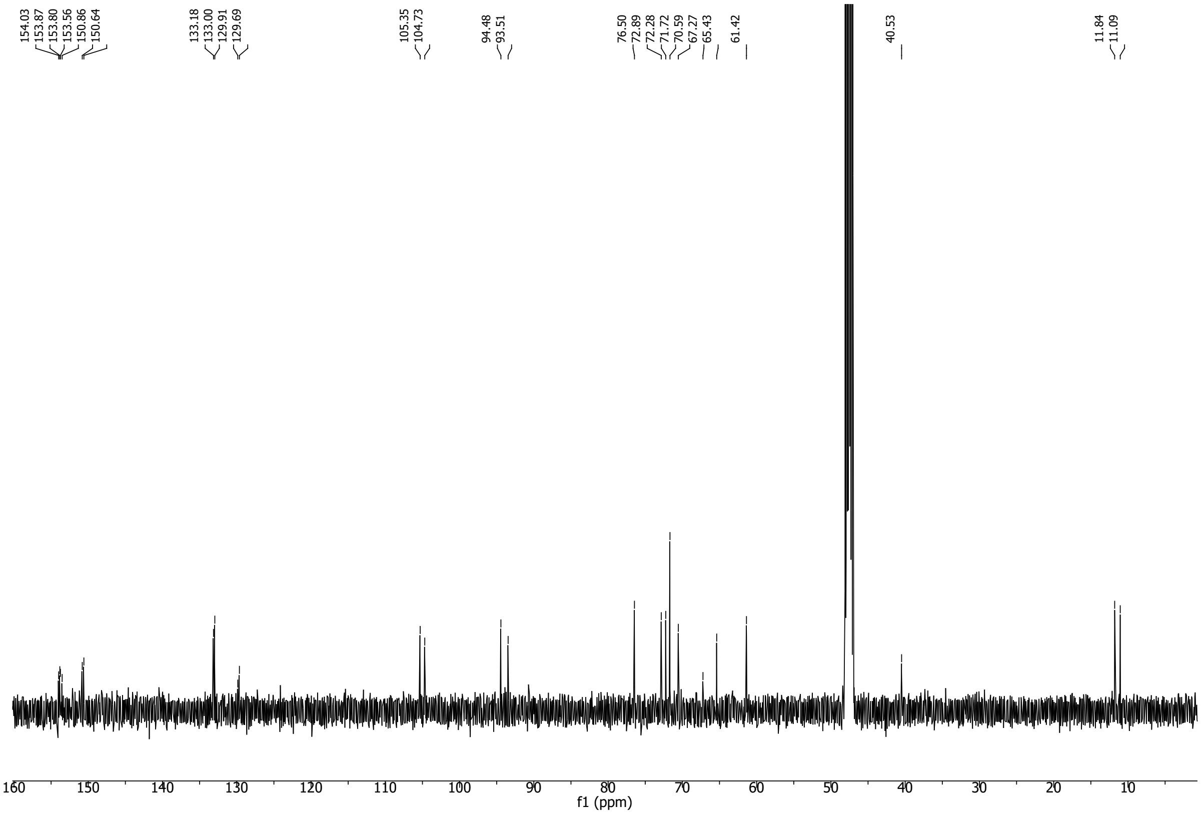

# Compound **15**

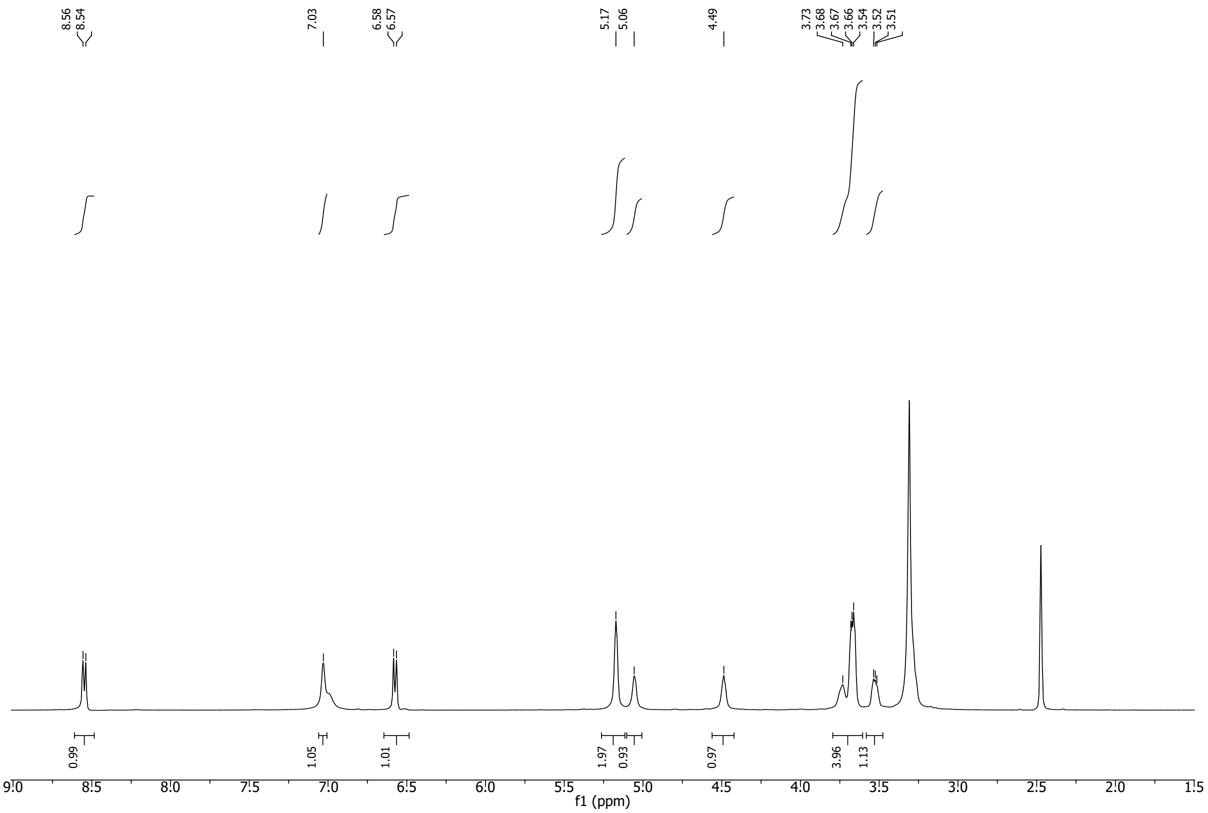
8.56
8.54
7.03
6.58
6.57
5.17
5.06
4.49
3.73
3.68
3.67
3.66
3.54
3.52
3.51
0.99
1.05
1.01
1.97
0.93
0.97
3.96
1.13
9.0 8.5 8.0 7.5 7.0 6.5 6.0 5.5 5.0 4.5 4.0 3.5 3.0 2.5 2.0 1.5
f1 (ppm)

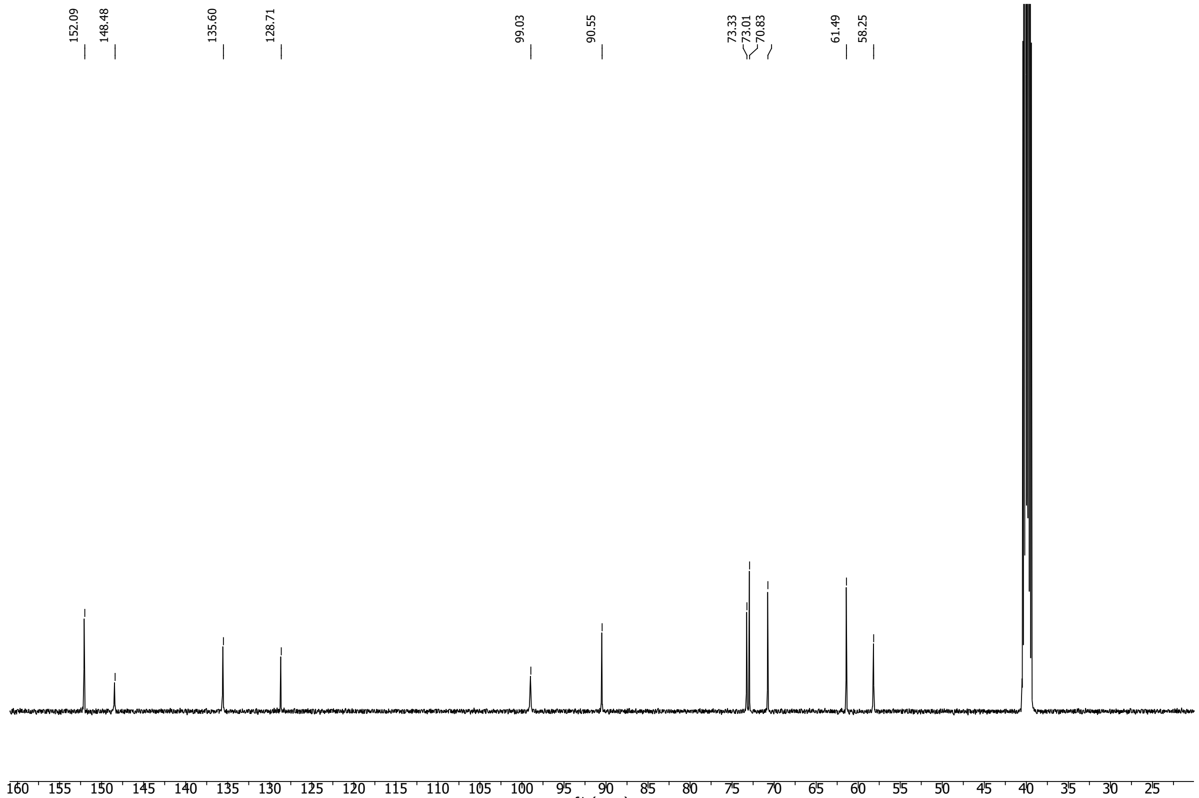
152.09
148.48
135.60
128.71
99.03
90.55
73.33
73.01
70.83
61.49
58.25
160 155 150 145 140 135 130 125 120 115 110 105 100 95 90 85 80 75 70 65 60 55 50 45 40 35 30 25
f1 (ppm)

Compound **16**

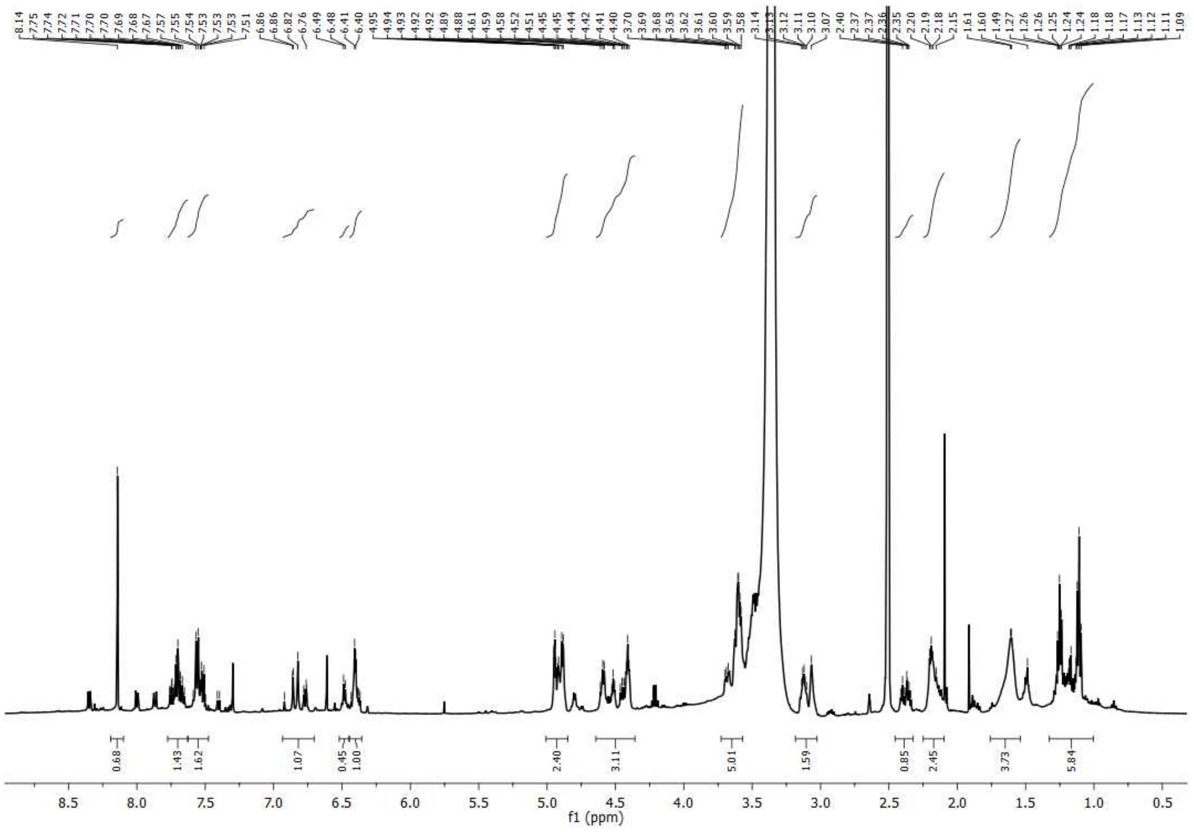
8.14 7.75 7.74 7.72 7.71 7.70 7.70 7.69 7.68 7.67 7.57 7.55 7.54 7.53 7.53 7.53 7.51 6.86 6.86 6.82 6.76 6.49 6.48 6.41 6.40 4.95 4.94 4.93 4.92 4.92 4.89 4.88 4.61 4.59 4.58 4.52 4.51 4.45 4.45 4.44 4.42 4.41 4.40 3.70 3.69 3.68 3.63 3.62 3.61 3.60 3.59 3.58 3.14 3.13 3.12 3.11 3.10 3.07 2.40 2.37 2.37 2.36 2.35 2.20 2.19 2.18 2.15 1.61 1.60 1.49 1.27 1.26 1.25 1.24 1.24 1.18 1.18 1.17 1.13 1.12 1.11 1.09
0.68 1.43 1.62 1.07 0.45 1.00 2.40 3.11 5.01 1.59 0.85 2.45 3.73 5.84
f1 (ppm)

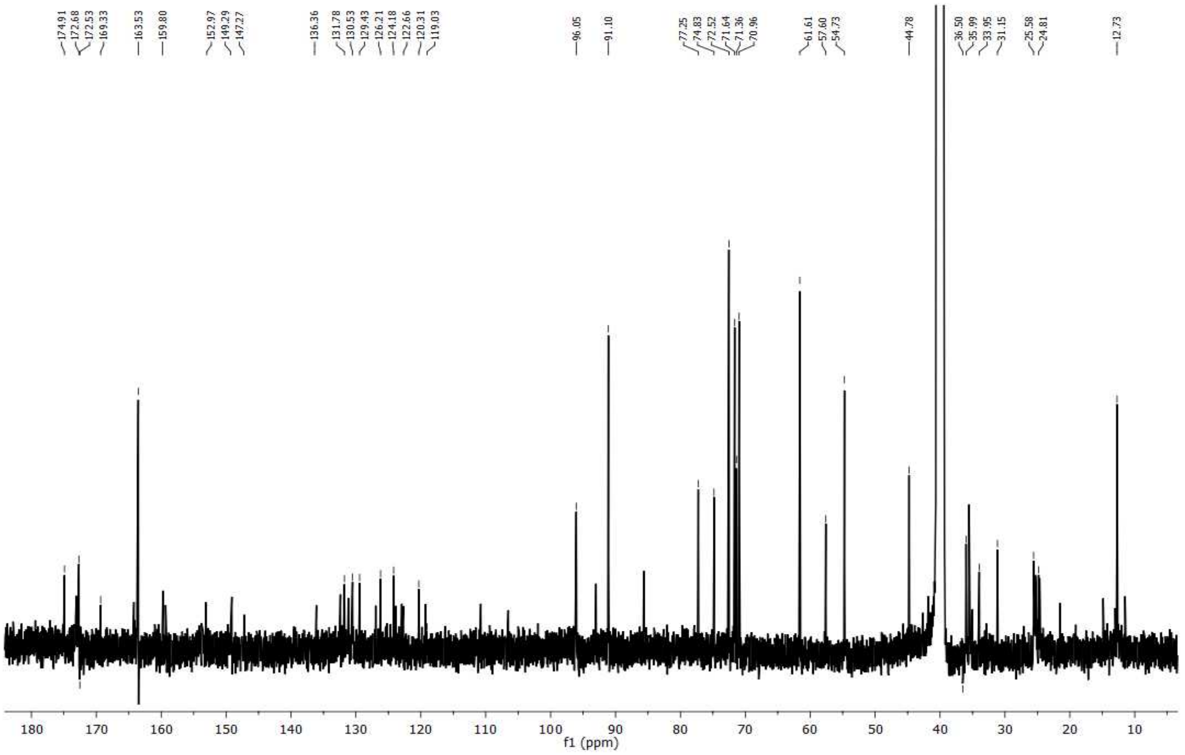
174.91 172.68 172.53 169.33 163.53 159.80 152.97 149.29 147.27 136.36 131.78 130.53 129.43 126.21 124.18 122.66 120.31 119.03 96.05 91.10 77.25 74.83 72.52 71.64 71.36 70.96 61.61 57.60 54.73 44.78 36.50 35.99 33.95 31.15 25.58 24.81 12.73
f1 (ppm)

Compound **pre-17**

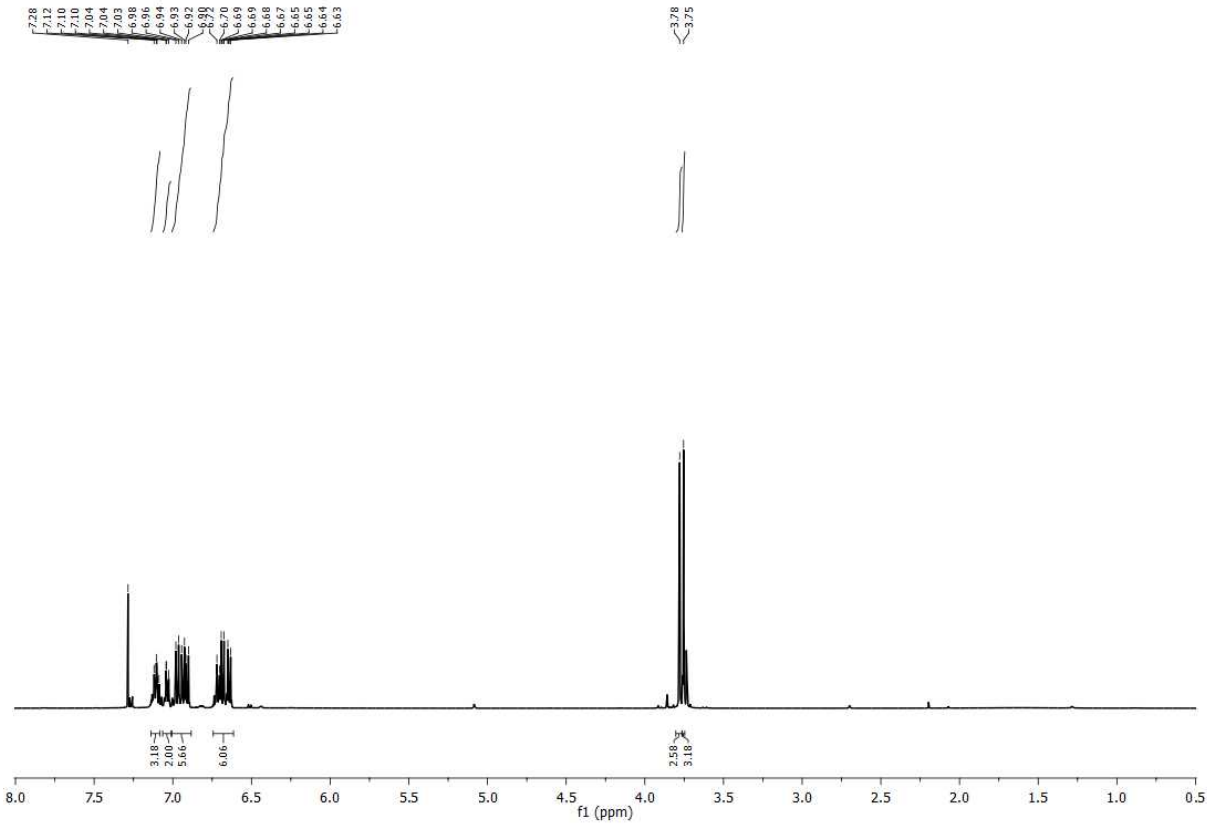

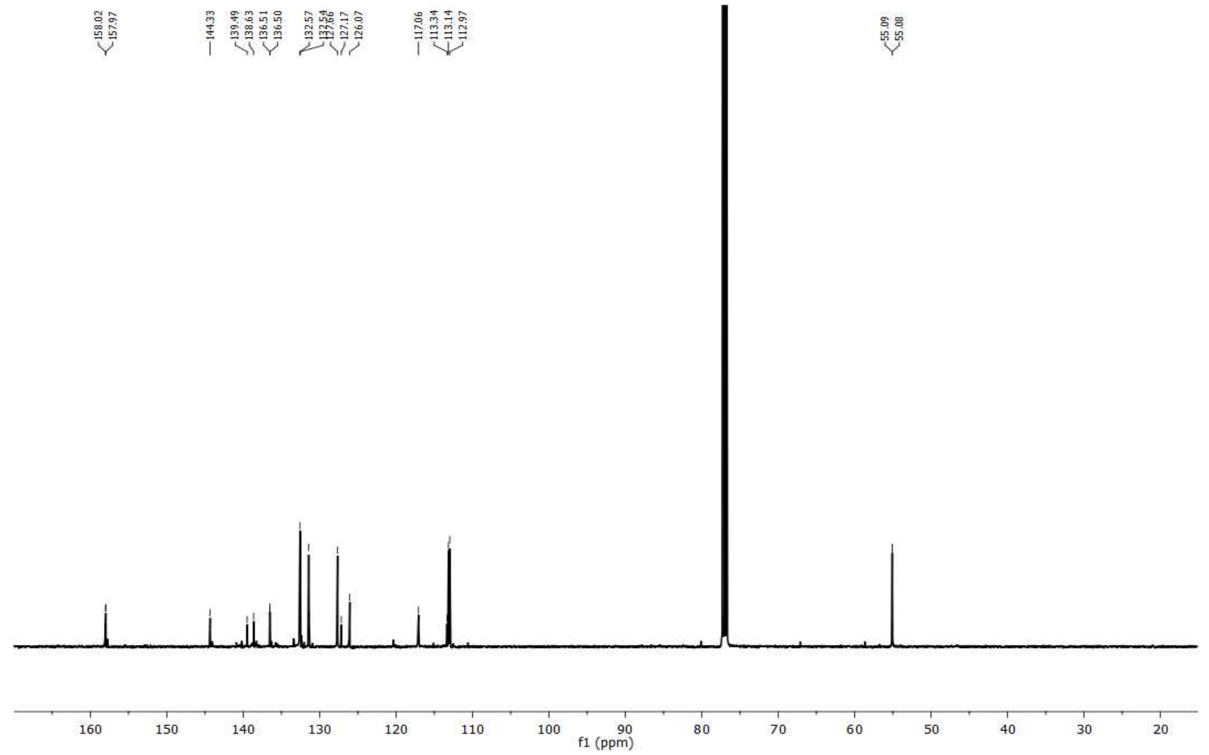

Compound **17**

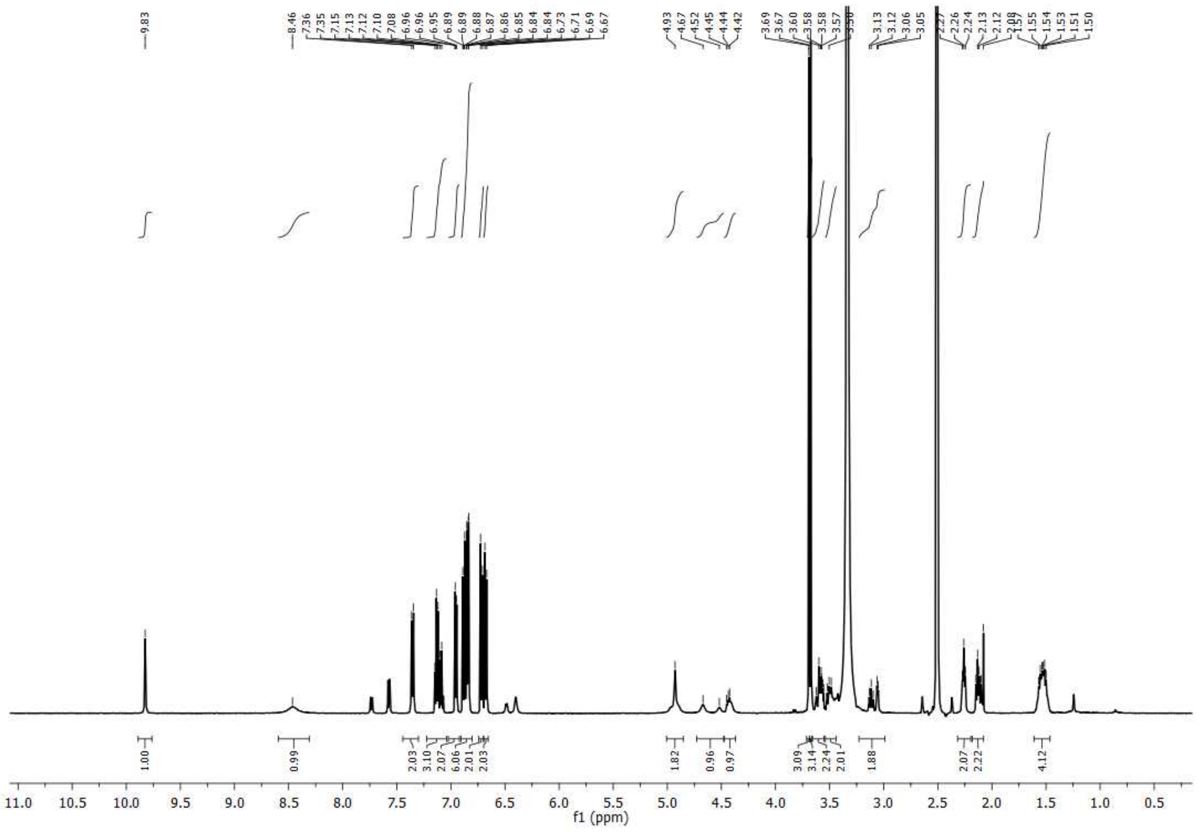
9.83
8.46 7.36 7.35 7.15 7.13 7.12 7.10 7.08 6.96 6.96 6.95 6.89 6.89 6.88 6.87 6.86 6.85 6.84 6.84 6.73 6.71 6.69 6.67
4.93 4.67 4.52 4.45 4.44 4.42
3.69 3.67 3.60 3.58 3.58 3.57 3.56
3.13 3.12 3.06 3.05
2.27 2.26 2.24 2.13 2.12 2.08 1.57 1.55 1.54 1.53 1.51 1.50
1.00 0.99 2.03 3.10 2.07 6.06 2.01 2.03 1.82 0.96 0.97 3.09 3.14 2.24 2.01 1.88 2.07 2.22 4.12
11.0 10.5 10.0 9.5 9.0 8.5 8.0 7.5 7.0 6.5 6.0 5.5 5.0 4.5 4.0 3.5 3.0 2.5 2.0 1.5 1.0 0.5
f1 (ppm)

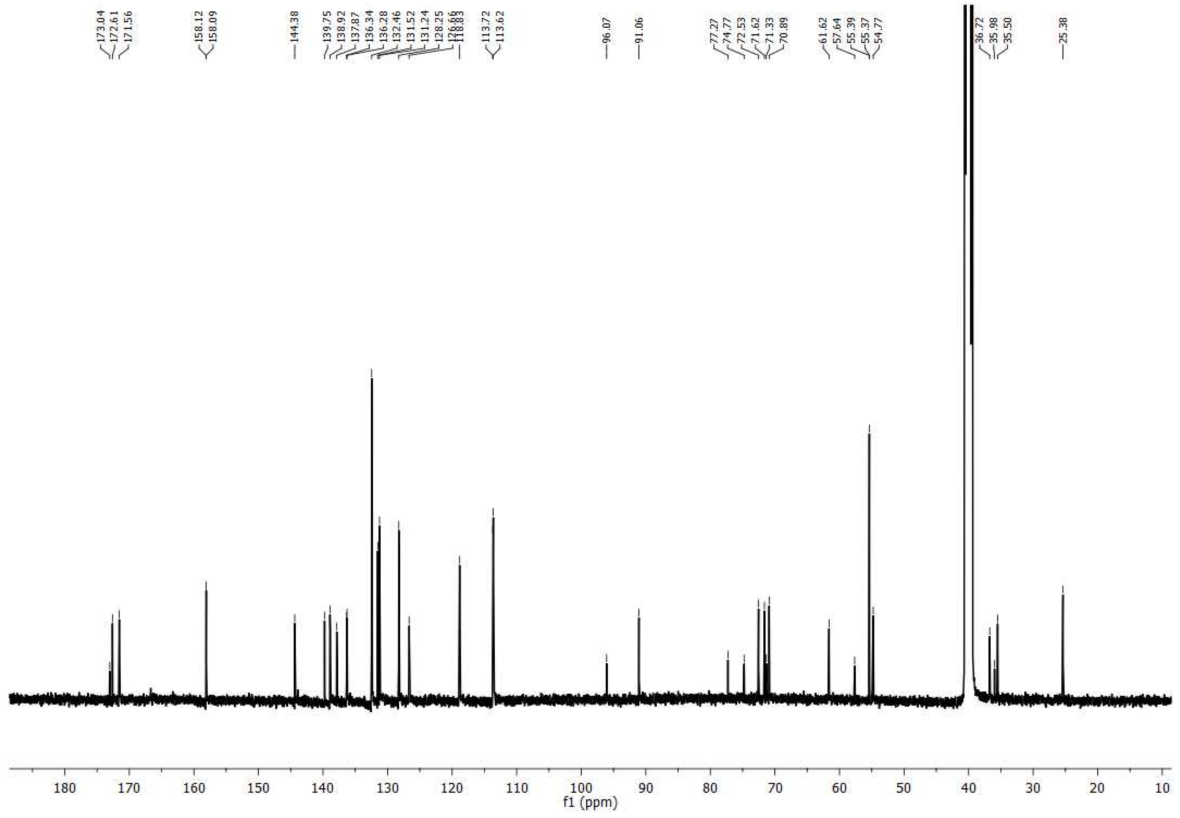
173.04 172.61 171.56
158.12 158.09
144.38
139.75 138.92 137.87 136.34 136.28 132.46 131.52 131.24 128.25 126.66 118.83
113.72 113.62
96.07
91.06
77.27 74.77 72.53 71.62 71.33 70.89
61.62 57.64 55.39 55.37 54.77
36.72 35.98 35.50
25.38
180 170 160 150 140 130 120 110 100 90 80 70 60 50 40 30 20 10
f1 (ppm)

**HPLC traces**

Compound **14**

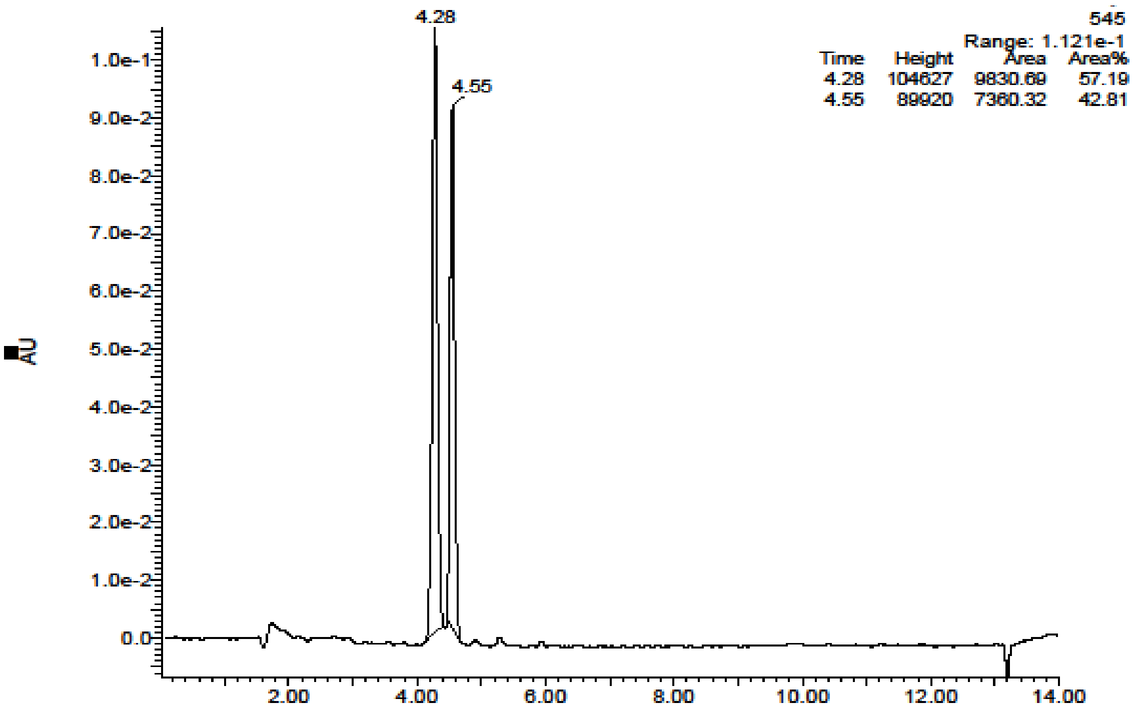


Compound **15**

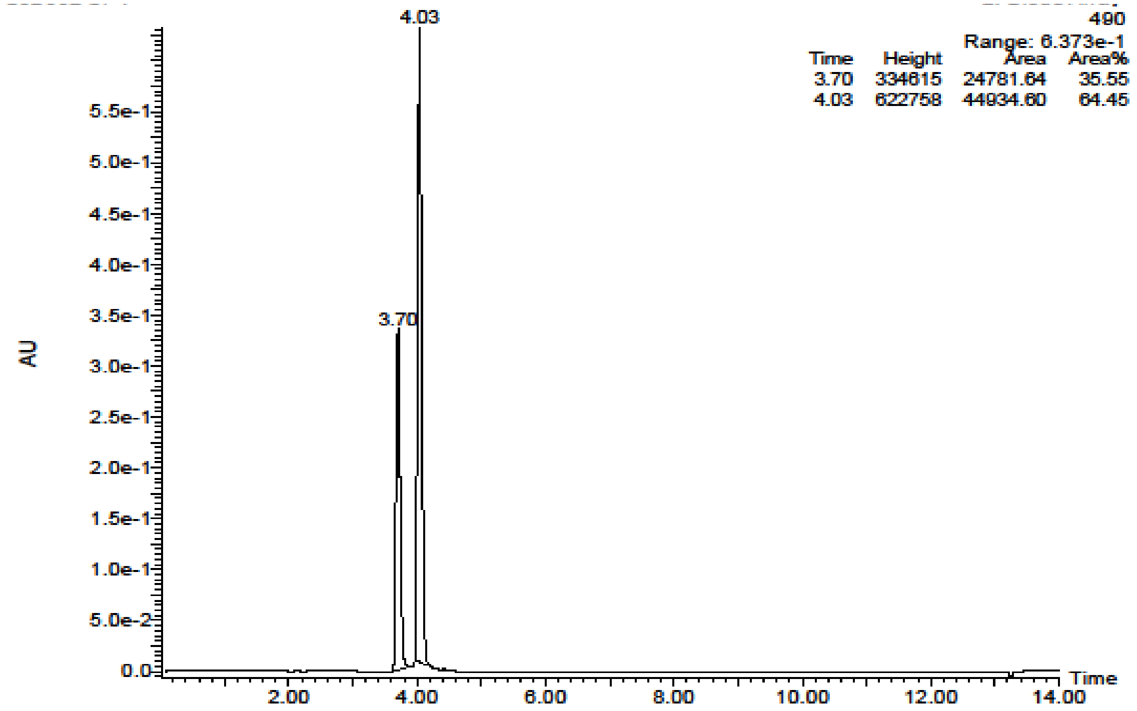

## Supplementary References


1. Kuru, E. et al. In situ probing of newly synthesized peptidoglycan in live bacteria with fluorescent D-amino acids. *Angew. Chem. Int. Ed.* **51**, 12519–12523 (2012).
2. Redmond, R. & Gamblin, J. N. A compilation of singlet oxygen yields from biologically relevant molecules. *Photochem. Photobiol.* **70**, 391–475 (1999).
3. Zhang Y. H. et al. AIE based GSH activatable photosensitizer for imaging-guided photodynamic therapy. *Chem. Commun.* **56**, 10317–10320 (2020).
4. Benson, S. et al. SCOTfluors: Small, conjugatable, orthogonal and tunable fluorophores for in vivo imaging of cell metabolism. *Angew. Chem. Int. Ed.* **58**, 6911–6915 (2019).
5. Liberek, B. et al. *N*-alkyl derivatives of 2-amino-2-deoxy-D-glucose. *Carbohydr. Res.* **340**, 1876–1884 (2005).
6. Zhang, S. et al. A maleimide-functionalized tetraphenylethene for measuring and imaging unfolded proteins in cells. *Chem. Asian J.* **14**, 904–909 (2019).
