# Supplementary material for: Photoactivatable metabolic warheads enable precise and safe ablation of target cells in vivo
Source: Nat Commun. 2021 Apr 22;12:2369. doi: 10.1038/s41467-021-22578-2 (PMC8062536; doi:10.1038/s41467-021-22578-2)
Supplement: Supplementary file 3 — Description of Additional Supplementary Files [file 41467_2021_22578_MOESM3_ESM.pdf]

Description of Additional Supplementary Files

File Name: Supplementary Movie 1
Description: **Recording of swimming motion of a representative untreated 3 dpf zebrafish larva.** Larvae were placed in a 10-cm Petri dish filled with Danieau’s solution. The steady-state swimming behavior was recorded using a SONY ExwaveHAD camera and EthoVision XT 7.0 software was used to analyze the recording and generate the tracking data.

File Name: Supplementary Movie 2
Description: **Recording of swimming motion of a representative 3 dpf zebrafish larva after treatment with compound 15 and light irradiation.** Larvae were placed in a 10-cm Petri dish filled with Danieau’s solution. The steady-state swimming behavior was recorded using a SONY ExwaveHAD camera and EthoVision XT 7.0 software was used to analyze the recording and generate the tracking data.

File Name: Supplementary Movie 3
Description: **Heartbeat monitoring of a representative untreated 3 dpf zebrafish larva.** Larvae were anesthetized with MS222 and mounted sideways in 3% methylcellulose for continuous heartbeat recording using a Hamamatsu camera (40 frames per second) and HCImage 4.2.0.33 software.

File Name: Supplementary Movie 4
Description: **Heartbeat monitoring of a representative 3 dpf zebrafish larva after treatment with compound 15 and light irradiation.** Larvae were anesthetized with MS222 and mounted sideways in 3% methylcellulose for continuous heartbeat recording using a Hamamatsu camera (40 frames per second) and HCImage 4.2.0.33 software.
