# Supplementary material for: Photoactivatable metabolic warheads enable precise and safe ablation of target cells in vivo
Source: Nat Commun. 2021 Apr 22;12:2369. doi: 10.1038/s41467-021-22578-2 (PMC8062536; doi:10.1038/s41467-021-22578-2)
Supplement: Supplementary file 8 — Reporting Summary [file 41467_2021_22578_MOESM8_ESM.pdf]

nature research

Corresponding author(s): Marc Vendrell
Last updated by author(s): Mar 11, 2021

Reporting Summary

Nature Research wishes to improve the reproducibility of the work that we publish. This form provides structure for consistency and transparency in reporting. For further information on Nature Research policies, see Authors & Referees and the Editorial Policy Checklist.

Statistics

For all statistical analyses, confirm that the following items are present in the figure legend, table legend, main text, or Methods section.

n/a	Confirmed	
☐	☒	The exact sample size (*n*) for each experimental group/condition, given as a discrete number and unit of measurement
☐	☒	A statement on whether measurements were taken from distinct samples or whether the same sample was measured repeatedly
☐	☒	The statistical test(s) used AND whether they are one- or two-sided<br>*Only common tests should be described solely by name; describe more complex techniques in the Methods section.*
☒	☐	A description of all covariates tested
☒	☐	A description of any assumptions or corrections, such as tests of normality and adjustment for multiple comparisons
☐	☒	A full description of the statistical parameters including central tendency (e.g. means) or other basic estimates (e.g. regression coefficient) AND variation (e.g. standard deviation) or associated estimates of uncertainty (e.g. confidence intervals)
☐	☒	For null hypothesis testing, the test statistic (e.g. *F*, *t*, *r*) with confidence intervals, effect sizes, degrees of freedom and *P* value noted<br>*Give P values as exact values whenever suitable.*
☒	☐	For Bayesian analysis, information on the choice of priors and Markov chain Monte Carlo settings
☒	☐	For hierarchical and complex designs, identification of the appropriate level for tests and full reporting of outcomes
☒	☐	Estimates of effect sizes (e.g. Cohen's *d*, Pearson's *r*), indicating how they were calculated

Our web collection on statistics for biologists contains articles on many of the points above.

Software and code

Policy information about availability of computer code

Data collection	Standard commercial softwares were used for data collection.<br>Specifically, HPLC-MS: Masslynx 4.1; BD 5Laser LSR and BD 5Laser FACSAria: BD FACSDIVA V8.0; Leica SP8: Leica Application Suite X; Synergy HT spectrophotometer: Gen 5; Nikon Ti2 Eclipse and Andor Zyla sCMOS: NIS element AR; Molecular Devices SpectraMax: SoftMax Pro GxP; DFT calculations: Gaussian09 D.01.
Data analysis	Data analysis, including statistical analysis, was performed using Graphpad Prism 6. FlowJo V10 was used to analyze flow cytometry data collected using the 5L LSR. Fluorescence image analysis was performed with Fiji ImageJ 1.52b. Zebrafish movie data was analyzed with EthoVision XT 7.0 and HCImage 4.2.0.33.

For manuscripts utilizing custom algorithms or software that are central to the research but not yet described in published literature, software must be made available to editors/reviewers. We strongly encourage code deposition in a community repository (e.g. GitHub). See the Nature Research guidelines for submitting code & software for further information.

Data

Policy information about availability of data

All manuscripts must include a data availability statement. This statement should provide the following information, where applicable:

- Accession codes, unique identifiers, or web links for publicly available datasets
- A list of figures that have associated raw data
- A description of any restrictions on data availability

The source data underlying Figs. 1b, 2c, 3c, 4b, c, 5e, 6b, d and Supplementary Figs. 2 and 4 are provided as a Source data file. Additional data that support findings of this study are available from the corresponding authors upon reasonable request. Source data are provided with this paper.

Field-specific reporting

Please select the one below that is the best fit for your research. If you are not sure, read the appropriate sections before making your selection.

☒ Life sciences ☐ Behavioural & social sciences ☐ Ecological, evolutionary & environmental sciences

For a reference copy of the document with all sections, see nature.com/documents/nr-reporting-summary-flat.pdf

Life sciences study design

All studies must disclose on these points even when the disclosure is negative.

Sample size	Unless stated, the minimum number of experimental repeats was 3, in line with current standards in the field. For experiments in animals, the sample size was calculated based on published experimental findings. Using an unpaired two sample t-test with a two-sided alternative hypothesis and a critical p-value of 0.5, we calculated that we would need at least 5 animals to be able to confirm a difference in the numbers of U87-nlsCrimson cells between the treatments with over 90% power.
Data exclusions	No data was excluded.
Replication	For key findings, at least 3 independent experiments were performed in order to check the reproducibility of results. These experiments include photophysical determinations (i.e. singlet oxygen quantum yields), in vitro experiments with U87 cells and in vivo experiments in zebrafish. All attempts of replication were successful and included into statistical analysis. For experiments in zebrafish, at least 5 animals were used per treatment group as justified in our sample size summary.
Randomization	Zebrafish larvae were randomly assigned to the different experimental groups. No randomization method was used for the other experiments because there were no other environmental variations and the experimental conditions were tightly controlled.
Blinding	Researchers were blinded for the quantification of fluorescent U87-nlsCrimson cells in zebrafish using ImageJ. Blinding was not carried out in other experiments as all comparisons were made using quantitative analysis of computational biochemical or cellular data.

Reporting for specific materials, systems and methods

We require information from authors about some types of materials, experimental systems and methods used in many studies. Here, indicate whether each material, system or method listed is relevant to your study. If you are not sure if a list item applies to your research, read the appropriate section before selecting a response.

Materials & experimental systems

n/a	Involved in the study	
☒	☐	Antibodies
☐	☒	Eukaryotic cell lines
☒	☐	Palaeontology
☐	☒	Animals and other organisms
☒	☐	Human research participants
☒	☐	Clinical data

Methods

n/a	Involved in the study	
☒	☐	ChIP-seq
☐	☒	Flow cytometry
☒	☐	MRI-based neuroimaging

Eukaryotic cell lines

Policy information about cell lines

Cell line source(s)	U87 and HCN2 cell lines were purchased from ATCC. U87-nlsCrimson were provided by Dr Dirk Sieger (University of Edinburgh).
Authentication	U87-nlsCrimson glioblastoma cells were confirmed as having the capacity to form 3D spheroids in vitro and microtumors in zebrafish larvae in vivo. U87 and HCN2 cell lines were authenticated by the vendor, ATCC, which uses morphology, karyotyping and PCR based approaches to confirm the identity of cell lines and to rule out both intra- and interspecies contamination.
Mycoplasma contamination	All cell lines tested negative for mycoplasma contamination.
Commonly misidentified lines (See ICLAC register)	No commonly misidentified cells were used in this study.

Animals and other organisms

Policy information about studies involving animals; ARRIVE guidelines recommended for reporting animal research

Laboratory animals	Species: Danio rerio. Age: 48 hpf (hours post-fertilization).
Wild animals	The study did not involve wild animals.
Field-collected samples	The study did not involve samples collected from the field.
Ethics oversight	Animal experimentation was approved by the ethical review committee of the University of Edinburgh and the UK Home Office. No project license was needed for the experiments in zebrafish because larvae were younger than 5 days old, in accordance with the Scientific Procedure Act 1986.

Note that full information on the approval of the study protocol must also be provided in the manuscript.

Flow Cytometry

Plots

Confirm that:

☒ The axis labels state the marker and fluorochrome used (e.g. CD4-FITC).

☒ The axis scales are clearly visible. Include numbers along axes only for bottom left plot of group (a 'group' is an analysis of identical markers).

☒ All plots are contour plots with outliers or pseudocolor plots.

☒ A numerical value for number of cells or percentage (with statistics) is provided.

Methodology

Sample preparation	U87 cells were incubated in glucose-free KRB buffer including or not compound 15 for 1 h. Cells were then illuminated or not with visible light (10 mW). Following incubation in supplemented DMEM for 16 h, cells were detached and re-suspended in KRB buffer to be analyzed by flow cytometry using Annexin V-Pacific Blue (4 µg mL-1) as an apoptosis marker.
Instrument	BD 5Laser LSR
Software	Data collection was performed with FACSDIVA V8.0 software. Data was analyzed with FlowJo V10 software.
Cell population abundance	For flow cytometry experiments, 10,000 events were acquired for every population of interest.
Gating strategy	Firstly, singlet cells were gated in FSC-A and FSC-H plots to exclude debris and non-singlet events. Secondly, gates were constructed based on laser scatter properties (FSC-A and SSC-A).

☒ Tick this box to confirm that a figure exemplifying the gating strategy is provided in the Supplementary Information.
